# Genomic insights and anti-phytopathogenic potential of siderophore metabolome of endolithic *Nocardia mangyaensis* NH1

**DOI:** 10.1038/s41598-024-54095-9

**Published:** 2024-03-07

**Authors:** Irina V. Khilyas, Maria I. Markelova, Liia R. Valeeva, Tatiana M. Ivoilova, Elena Shagimardanova, Alexander V. Laikov, Anna A. Elistratova, Ekaterina S. Berkutova, Guenter Lochnit, Margarita R. Sharipova

**Affiliations:** 1 Institute of Fundamental Medicine and Biology, Kazan (Volga Region) Federal University, Kazan, Russian Federation; 2 Laboratory of Multiomics Technologies of Living Systems, Institute Fundamental Medicine and Biology, Kazan (Volga Region) Federal University, Kazan, Russian Federation; 3 Skolkovo Institute of Science and Technology, Moscow, Russian Federation; 4 Life Improvement by Future Technologies (LIFT) Center, Moscow, Russian Federation; 5 Protein Analytics, Institute of Biochemistry, Faculty of Medicine, Justus Liebig University Giessen, Giessen, Germany

**Keywords:** Endolithic bacteria, *Nocardia*, Siderophores, Metabolome, NRPS, Antifungal activity, Biocontrol agent, Biotechnology, Microbiology, Plant sciences

## Abstract

*Actinobacteria* are one of the predominant groups that successfully colonize and survive in various aquatic, terrestrial and rhizhospheric ecosystems. Among actinobacteria, *Nocardia* is one of the most important agricultural and industrial bacteria. Screening and isolation of *Nocardia* related bacteria from extreme habitats such as endolithic environments are beneficial for practical applications in agricultural and environmental biotechnology. In this work, bioinformatics analysis revealed that a novel strain *Nocardia mangyaensis* NH1 has the capacity to produce structurally varied bioactive compounds, which encoded by non-ribosomal peptide synthases (NRPS), polyketide synthase (PKS), and post-translationally modified peptides (RiPPs). Among NRPS, five gene clusters have a sequence homology with clusters encoding for siderophore synthesis. We also show that *N. mangyaensis* NH1 accumulates both catechol- and hydroxamate-type siderophores simultaneously under iron-deficient conditions. Untargeted LC–MS/MS analysis revealed a variety of metabolites, including siderophores, lipopeptides, cyclic peptides, and indole-3-acetic acid (IAA) in the culture medium of *N. mangyaensis* NH1 grown under iron deficiency. We demonstrate that four CAS (chrome azurol S)-positive fractions display variable affinity to metals, with a high Fe^3+^ chelating capability. Additionally, three of these fractions exhibit antioxidant activity. A combination of iron scavenging metabolites produced by *N. mangyaensis* NH1 showed antifungal activity against several plant pathogenic fungi. We have shown that the pure culture of *N. mangyaensis* NH1 and its metabolites have no adverse impact on *Arabidopsis* seedlings. The ability of *N. mangyaensis* NH1 to produce siderophores with antifungal, metal-chelating, and antioxidant properties, when supplemented with phytohormones, has the potential to improve the release of macro- and micronutrients, increase soil fertility, promote plant growth and development, and enable the production of biofertilizers across diverse soil systems.

## Introduction

Modern agriculture confronts a range of complex challenges, including the degradation and exhaustion of agricultural land, the loss of biodiversity, the rise of phytopathogenic diseases and reduced crop productivity. Plants on degraded land suffer from elevated levels of salinity, drought and heavy metals, leading to stress and impaired growth. Iron deficiency is also a frequent occurrence in agricultural soil, in addition to adverse conditions^1^. Synthetic fertilisers are a beneficial approach to restoring degraded land and promoting plant nutrition^2^. However, they do not enhance soil fertility or microbial biodiversity despite their effectiveness in improving plant growth. Moreover, the high concentration of exogenous fertilizers reduces the solubility and availability of iron to plants in iron-deficient soils. An environmentally-friendly substitute is the application of organic fertilizers supplemented with plant growth-promoting (PGP) microorganisms^3^. PGP bacteria produce iron-chelating compounds (siderophores) that reduce iron deficiency and improve physiological and biochemical processes in plants in degraded soils. For agricultural applications, especially in organic farming systems, it is essential to screen microbial biological control agents from different habitats. Extreme environments provide an ecological niche for discovering novel microorganisms with immense metabolic potential, especially specialized secondary metabolites.

Actinobacteria are highly adaptable to different habitats and are used as biostimulants due to their ability to synthesize secondary metabolites, bioactive compounds, enzymes, plant phytohormones and agroactive chemicals^4^. In addition, *Actinomycetes* species have been found to increase the availability of essential metals in drylands and degraded soils, improve stress tolerance in plants, and protect against phytopathogens, ultimately benefiting major crop yields^5^. They are a prevalent bacterial group in rhizospheric environments, providing soluble phosphate to plants, fixing nitrogen, and producing 1-aminocyclopropane-1-carboxylate (ACC) deaminase along with phytohormones such as gibberellin, cytokinin, and indole acetic acid (IAA)^5^. *Actinomycetes* producing siderophores improve nutrient absorption in deteriorated agricultural soils, resulting in promotion of respiration, photosynthesis, and plant growth^1^.

Among all actinobacteria, *Nocardia* species are widely distributed in different aquatic, terrestrial and rhizhospheric ecosystems^6–8^. Morphologically, members of the *Nocardia* genus display distinct characteristics, such as the formation of fragmentary hyphae, short-chain spores, and pigmented rough or smooth irregular colonies^6,9^. Furthermore, the genomic features of *Nocardia* spp. possess valuable insights regarding the pool of biosynthetic novel natural products that may be useful in agriculture, especially for soil fertility, plant health maintenance, biofertilisers and biopesticides^10^. The genome of *Nocardia* strains contains NRPS, PKS or hybrid NRPS-PKS gene clusters, which are linked to the synthesis of a diverse array of secondary metabolites^11–13^.

NRPS genes synthesise siderophores, which act as natural ligands for the binding of iron. These facilitate the dissolution, sequestration and transport of iron from the environment, making it available to microorganisms or plants^14^. Bacterial siderophores bind iron and perform various functions in agriculture and the environment, such as hindering plant phytopathogens, improving nutrient absorption, and promoting plant growth^15^. *Actinobacteria* are widely known producers of siderophores, which can be distinguished according to their functional moieties, such as catecholate, hydroxamate, carboxylate and mixed types^16^. To combat iron deficiency, *Nocardia* species synthesise various siderophores, including nocobactins, nocardichelins, formobactin, amamistatins, asterobactin, brasilibactin, and nocardimicins^17–23^. The compounds studied have mainly shown antibacterial, cytotoxic, antineoplastic and antioxidant properties^9,11^. The aim of the present study was to determine the taxonomic classification of strain NH1 isolated from endolithic environments, and to investigate its biosynthetic capabilities and siderophore diversity in order to discover its agrobiotechnological and ecological potential.

## Results

### Physiological and genomic features of Nocardia mangyaensis NH1

The NH1 strain is a Gram-positive, aerobic, non-motile, catalase-positive, road-shaped bacterium (0.5–0.7 μm wide, 1–2 μm long) isolated from hydromagnesite found in the vent of the serpentinite rock (Khalilovsky massif, Russia)^24^. The NH1 strain was obtained from a colony on Luria agar (LA) by plating an aqueous rinse solution of crushed hydromagnesite rock onto the surface of the agar. It forms a substrate mycelium that is colored light orange or pink fragments into irregular rod-shaped elements. Among plant growth-promoting (PGP) activities, the secretion of siderophores and synthesis of phytohormones, such as indole acetic acid (IAA), have been determined.

**Figure 1:**
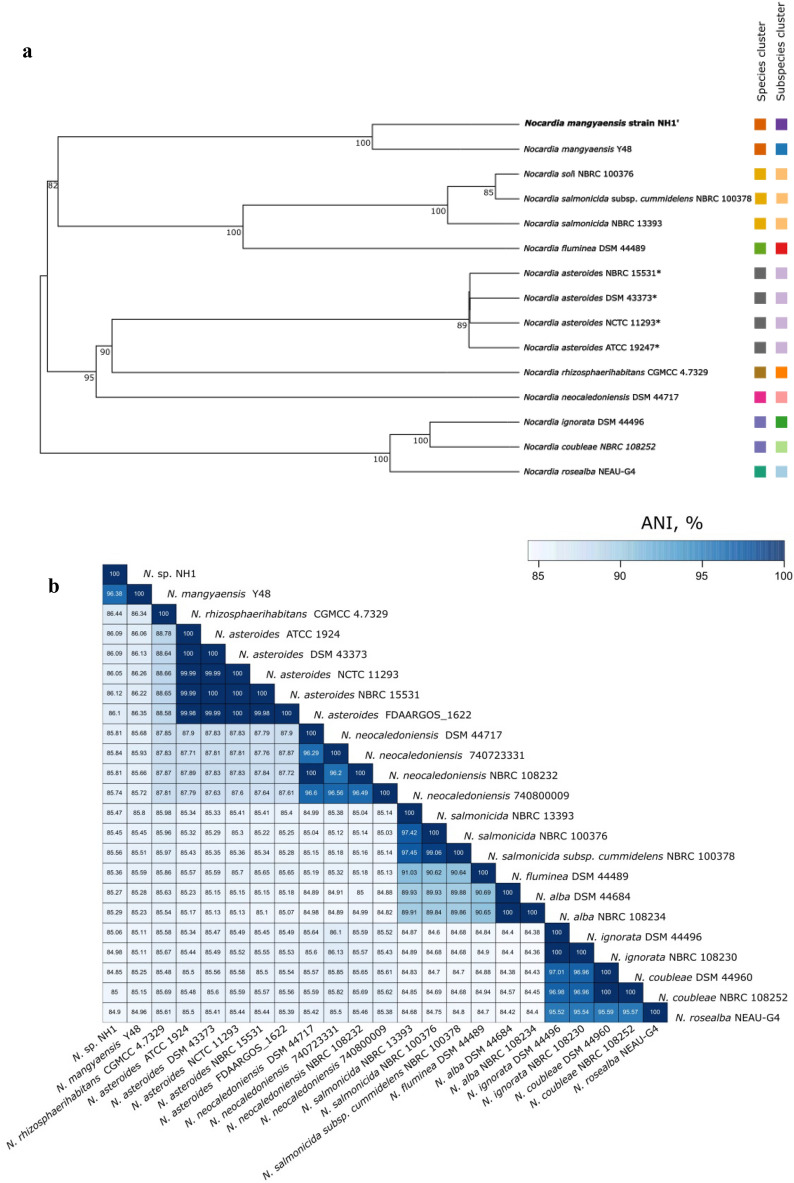
(**a**) A phylogenomic tree of the genus *Nocardia* based on in silico DNA-DNA hybridization (DDH) comparisons. The branch lengths are scaled in terms of GBDP distance formula *d*_*5*_. The numbers above branches are GBDP pseudo-bootstrap support values > 60% from 100 replications, with an average branch support of 81.3%. The tree was rooted at the midpoint. (**b**) Heat map of pairwise average nucleotide identity (ANI) among selected *Nocardia* genomes.

The size of a whole genome of strain NH1 is 6,743,715 bp. The DNA G + C content is 68%. The genomic analysis of NH1 resulted in a DNA-DNA hybridisation (DDH) value of 73.9%, demonstrating 100% similarity to *Nocardia mangyaensis* Y48 at the level of the subspecies cluster (Fig. 1a, see Supplementary Tables S1, S2). The ANI (average nucleotide identity) analysis showed that NH1 shares 96.38% nucleotide identity with *N. mangyaensis* Y48 isolated from oil contaminated soil, making it the closest specie^25^ (Fig. 1b). It also shares 86.44% similarity with *Nocardia rhizospaerihabitans* CGMCC 4.7329, *Nocardia asteroides* ATC 1924, DSM 43373, NCTC 11293 and NBRC 15531 (Fig. 1b, see Supplementary Table S1).

#### Specialized metabolite biosynthetic gene clusters

The mining of *N. mangyaensis* NH1 genome revealed a total of 11 NRPS gene clusters, including the predicted siderophore synthetase genes arranged across 5 clusters, 4 PKS and ribosomally-synthesized and posttranslationally-modified peptides (RiPPs) gene clusters. In addition to the predicted genes encoding the synthesis of siderophores, the genome of *N. mangyaensis* NH1 was found to contain a gene cluster responsible for the synthesis of aminopolycarboxylic acid metallophores, as well as an NRPS gene cluster involved in the synthesis of biosurfactants. Three adenylation domains of siderophore-like gene clusters 1.1, 6.1 and 14.1 had specificity for serine (Ser) suggesting the formation of catechol-type siderophores^26^ (Table 1). The transcription of two NRPSs and an aminopolycarboxylic acid metallophore is regulated by the iron-regulatory protein (DmdR1). The zinc uptake regulator (Zur), a member of the Fur (ferric uptake regulator) controls the transcription of three NRPSs. The nickel-responsive repressor (NuR) from the Fur family regulates the transcription of a single NRPS (Table 1). We found a association between siderophore-like gene clusters in *N. mangyaensis* NH1 and ABC (ATP-dependent pumps) and MFS (Major Facilitator Superfamily) transporters (Table 1). Additionally, the siderophore import protein FecCD was detected in clusters 1.1 and 6.2. The siderophore-reductase system-related transporter FhuF was identified in NRPS gene cluster 2.4 (Table 1).

**Table 1:** AntiSmash prediction of siderophore-like gene clusters in the genome of *N. mangyaensis* NH1.

Gene cluster	Type	Polymer prediction	Transcription factor binding site	Transporter genes
Cluster 1.1	NRPS, NRP-metallophore	(X − D − Ser) + (OH − Orn) + (D − X)	Fe II	ABC transporter, FecCD domain
Cluster 2.4	NRPS, NRP-metallophore, NI-siderophore*	(X)	Fe II	ABC transporter, Ferric iron reductase Fhu F-like transpoter
Cluster 6.1	NRPS, NRP-metallophore	(Ser − Cys − Cys) + (Cys)	Zn, Ni	ABC transporter, MFS
Cluster 6.2	NRPS, T1PKS**	(ccemal)	Zn	MFS, FecCD domain
Cluster 14.1	NRPS	(Ser − Leu − X − Leu)	Zn	ABC transporter
Cluster 7.2	Aminopolycarboxylic acid metallophore		Fe II	MATE

*NRPS-independent, IucA/IucC-like siderophores.

**Type I PKS (Polyketide synthase).

X—Domain that is predicted to be inactive.

KEGG was used to map the genes involved in the IAA synthesis pathway in the *N. mangyaensis* NH1 genome (see Supplementary Fig. S2). Two primary gene groups responsible for the synthesis of 3-indoleacetic acid have been identified. The former group contains 14 aldehyde dehydrogenase genes, whereas the latter group consists of only two aliphatic amidases (see Supplementary Fig. S2). The LC–MS/MS analysis has verified the accumulation of IAA (32 ± 2 µg/mL) by *N. mangyaensis* NH1 in M9 minimal medium under Fe-deficient conditions (see Supplementary Fig. S3C).

### Isolation and identification of siderophores from culture supernatant

Siderophore production by *N. mangyaensis* NH1 was evaluated using a chrome azurol S (CAS) agar plate assay. A clear zone around the bacterial colony was observed after 72 h, indicating the production of iron scavenging compounds (see Supplementary Fig. S5). *N. mangyaensis* NH1 was grown on M9 minimal medium with restricted iron supply to quantify the amount of catechol and hydroxamate type siderophores (see Supplementary Fig. S6). The Arnow test showed that a concentration of the catechol-type siderophores was reached 15.8 ± 2.9 μM after 72 h of growth. Similarly, the Atkin assay indicated the accumulation of hydroxamate siderophores to an amount of 87 ± 21 μM after 96 h of growth (see Supplementary Fig. S6). Accumulation of siderophores in the liquid medium occurred in a growth-phase-dependent manner of *N. mangyaensis* NH1. HPLC analysis detected four primary metabolites that were either absent or substantially suppressed in the presence of iron chloride III (see Supplementary Fig. S7).

To obtain a sufficient amount of iron-scavenging metabolites for screening of their activities, the bacterial culture of *N. mangyaensis* NH1 was scaled-up to a volume of 1L under conditions of limited iron, which promotes the production of siderophores. Metabolites were extracted from the culture supernatant after 72 h by using XAD16 resin. Untargeted LC–MS/MS analysis was used for the metabolic profiling of *N. mangyaensis* NH1 grown under iron-deficient conditions. Among the 1 553 unique metabolites with significant matches, the study detected the presence siderophores related to mycobactin (m/z 911.60, 897.58 and 869.55) and carboxymycobactin-like compounds (m/z 815.43, 773.38, 759.37, 829.45 and 897.58), which are produced by *Mycobacterium* (Table 2)^27^. The nocobactine-like compound (m/z 787.44) was identified in *N. mangyaensis* NH1 supernatant, commonly found in *Nocardia* species (Table 2)^12^. Additionally, siderophores produced by *N. mangyaensis* NH1 included loihichelin E from *Halomonas* sp. and desferribactin from *Pseudomonas fluorescens* ATCC 13525^28,29^.

**Table 2:** Siderophore-like compounds from the cultural liquid of *N. mangyaensis* NH1 annotated by the Global Natural Products Social Molecular Networking (GNPS) Moldiscovery.

Name of identified compound	Metabolite mass	m/z	RT	Adduct	Metabolite FDR* (%)
Carboxymycobactins-6	815.43	408.72	2241.25	M + 2H	0.00
Carboxymycobactin-3	773.38	387.69	2189.47	M + 2H	0.00
Carboxymycobactin_2	759.37	380.70	1571.62	M + 2H	0.12
	759.37	380.69	2255.09	M + 2H	0.10
Carboxymycobactin-7	829.45	415.73	1989.07	M + 2H	0.07
	897.58	449.80	2953.79	M + 2H	0.07
Mycobactin_(20,DB)	911.60	456.81	3029.24	M + 2H	0.12
Mycobactin_(19,DB)	897.58	449.80	2953.79	M + 2H	0.07
Mycobactin_P_(C18_alkyl)	869.55	435.79	2671.56	M + 2H	0.10
Loihichelin_E	1113.56	557.79	2386.23	M + 2H	0.00
Desferribactin_ATCC_13525	1177.57	589.79	1328.45	M + 2H	0.00
Nocobactin_NA	787.44	394.73	1374.45	M + 2H	0.08

*False discovery rate (FDR). The FDR below 0.9% was used as filters for processing of the final results: the highest quality annotations would require passing thresholds for both metrics.

The LC–MS/MS results revealed a variety of metabolites produced by *N. mangyaensis* NH1 in responce to Fe-limited conditions. These metabolites were identified as previously recognized lipopeptides such as bacillomycin (m/z 1034.53), agrastatin (m/z 1448.78), bacillopeptins (m/z 1034.53 and 1020.51), minutissamide (m/z 1151.57), and syringostatin (m/z 1194.59). These lipopeptides are commonly synthesized by representatives of the genera *Bacillus, Pseudomonas*, and *Anabena* (Table 3)^30–34^.

**Table 3:** Lipopeptide-like compounds from the cultural liquid of *N. mangyaensis* NH1 annotated by the GNPS DEREPLICATOR + of Classic Molecular Networking V2/Moldiscovery.

Name of identified compound	Metabolite mass	m/z	RT	Adduct	Metabolite FDR* (%)
Bacillomycin	1034.53	518.27	3756.36	M + 2H	0.00
Agrastatin_A	1448.78	725.40	4407.16	M + 2H	0.00
Bacillopeptin_B	1034.53	518.27	3792.26	M + 2H	0.00
Minutissamide_A_12ʹ-Chloro	1151.57	576.79	2192.62	M + 2H	0.00
Syringostatin B	1194.59	598.30	1830.76	M + 2H	0.00
Bacillopeptin-A	1020.51	511.26	3588.17	M + 2H	0.00

*False discovery rate (FDR). The FDR below 0.9% was used as filters for processing of the final results: the highest quality annotations would require passing thresholds for both metrics.

The numbers of the cyclic peptides related to mycobacillin (m/z 1527.55) and mycosubtilin (m/z 1198.63) produced by members of *Bacillus* spp.; as well as mycoplanecins (m/z 1196.78) and peptidolipins (m/z 963.66 and 1089.80) originated from *Actinoplanes* and *Nocardia* genera, were found in the supernatant of *N. mangyaensis* NH1, when cultured under Fe-limited conditions (Table 4)^35–38^.

**Table 4:** Cyclic peptide-like compounds from the cultural liquid of *N. mangyaensis* NH1 annotated by the GNPS Dereplicator.

Name of identified compound	p-value	Peptide mass	SpecMass	RT	Adduct	PSM FDR (%)
Mycobacillin	8.9e − 9	1527.55	774.39	2378.97	M + 2H	2.40
Mycoplanecin_D	1.1e − 9	1196.78	565.36	4047.87	M + 2H	2.17
Mycoplanecin_C	1.5e − 8	1196.78	411.93	3194.78	M + 3H	4.49
Mycosubtilin	8.9e − 7	1198.63	602.83	2376.23	M + 2H	2.50
Peptidolipin NA	6.7e − 14	963.66	502.74	1489.34	M + 2H	0.37
Peptidolipin B	4.4e − 12	189.80	502.74	1470.87	M + 2H	0.71

*Peptide-to-spectrum match (PSM) false discovery rate (FDR). Peptide FDR was maintained below 5% by requiring a minimum number of spectral counts for each peptide across the dataset.

**Figure 2:**
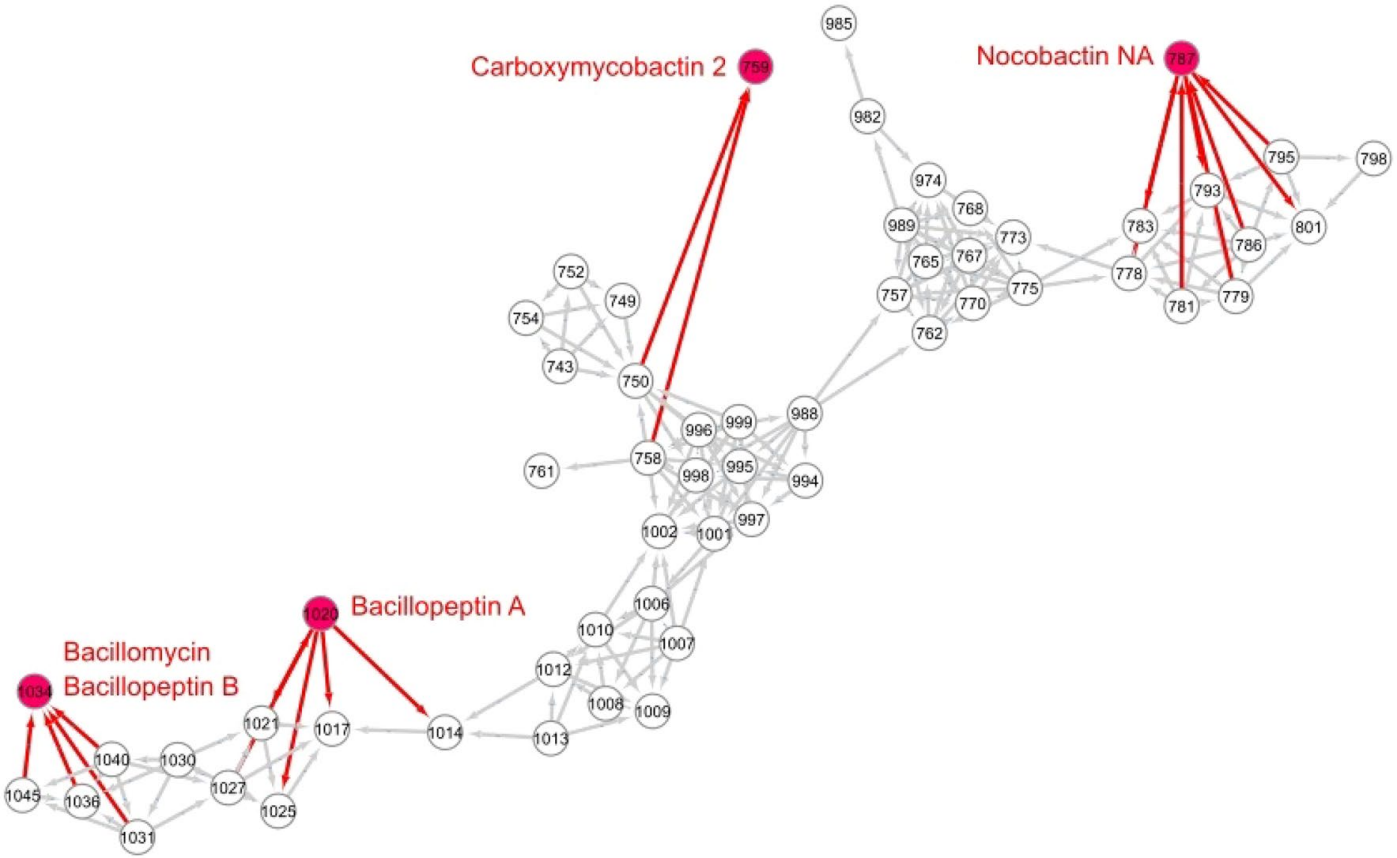
Molecular networking of siderophore- and lipopeptide-like compounds produced by *N. mangyaensis* NH1 under Fe-limited conditions. Each node represents a distinct metabolite that needed to be present in two biological replicates. The known siderophore- and lipopeptide-like compounds from *N. mangyaensis* NH1 are indicated as red circles. Four separate families in one cluster can be recognized and include nocobactine NA, carboxymycobactin 2, bacillomycin, bacillopeptin A and B. For this network, nodes were manually positioned, and only chosen edges are displayed to enhance clarity of presentation. The numerical values in the nodes indicate the corresponding m/z value of metabolites (rounded to one digit). The software Cytoscape was used for visualization.

Using molecular networking, metabolites with similar spectral features were grouped together. The resulting clusters were then visualized through Cytoscape software after exporting the outputs from GNPS-METABOLOMICS-SNETS-V2. The network of *N. mangyaensis* NH1 revealed one cluster combining siderophore- and lipopeptide-like compounds such as nocobactine NA, carboxymycobactin 2, bacillomycin, bacillopeptin A and B (Fig. 2). Metabolites in a given cluster are classified as belonging to the same molecular family and share analogous functions, particularly siderophore activity.

### Activity of siderophores

After preparative HPLC, four fractions were collected with retention times of 3, 9–10, 11, and 14 min. These fractions were then tested for antioxidant activities, metal-binding and CAS assay.

#### Screening for metal-chelating activity

Fraction 3 was characterized by the appearance of an absorbance peak at 320 nm. By contrast, after incubation with metals, no 320 nm peak was detected. Nonetheless, the spectrum has been altered and displays a peak between 240 and 250 nm solely in the existence of iron (Table 5, see Supplementary Fig. S8A). The absorbance spectrum of the metal-free fraction 9–10 revealed peaks at 280 and 330 nm. Alongside the iron-related peaks at 240 and 250 nm, we identified a Cu^2+^-related peak at 310 nm and Ga^3+^-related peaks at 300 and 310 nm (Table 5, see Supplementary Fig. S8B). Fraction 11 exhibited an absorbance peak at 250 and 310 nm in the absence of metal, with an iron-related peak at 240 and 250 nm. Additionally, a new absorbance peak at 280 nm was observed in the presence of Ga^3+^ (Table 5, see Supplementary Fig. S8C). Among all CAS-positive fractions, fraction 14 with a unique peak at 270 nm revealed spectral alterations in the presence of examined metals. A new absorption peak at 400 nm was detected for all these metals except for iron, which generates an iron-associated peak at 240 and 250 nm (Table 5, see Supplementary Fig. S8D).

**Table 5:** UV/Vis characteristics of CAS-positive HPLC-purified fractions of *N. mangyaensis* H1 in the presence of various metals.

Metals	Fraction 3	Fraction 9–10	Fraction 11	Fraction 14
No metal	320 nm	280, 330 nm	250, 310 nm	270 nm
Co^2+^	NM	NM	NM	400 nm
Al^3+^	NM	NM	NM	400 nm
Ni^2+^	NM	NM	NM	400 nm
Fe^3+^	240–250 nm	240–250 nm	240–250 nm	240, 250, 400 nm
Zn^2+^	NM	NM	NM	400 nm
Mn^2+^	NM	NM	NM	400 nm
Cu^2+^	NM	310 nm	NM	400 nm
Ga^3+^	NM	300, 310 nm	280 nm	400 nm

*NM—not modified. The UV/visible absorbance remains unaltered when compared to the spectrum of the metal-free fraction. Each of the fractions was dissolved in water.

#### Antioxidant activity

To determine the antioxidant activity, 2 µg/mL HPLC-purified CAS-positive fractions of *N. mangyaensis* NH1 were analysed using the AmplexRed assay based on hydrogen peroxide detection. The initial absorbance of a positive control (10 µM H_2_O_2_) started at 0.05 and progressively increased throughout the experiment (see Supplementary Fig. S9). The absorbance of fractions 3.0, 9.0–10.0 and 11.0 was lower than that of the positive control from the third minute of the experiment. The absorbance signal was remained stable for the next 3 h. A slight increase in absorbance was detected for fraction 14.0 compared to the positive control. The negative control was used water without H_2_O_2_ (see Supplementary Fig. S9).

#### Antifungal activity

A mixture of metabolites from *N. mangyaensis* NH1 grown under Fe-limited conditions was evaluated for its antifungal property against strong phytopathogenic strains including *Colletotrichum coccodes* MF 16-014, *Rhizoctonia solani* MFP 936011, *Fusarium oxisporum* PR 57 and *Alternaria* sp. obtained from potato tubers (Table 6, see Supplementary Fig. S10)^39^. The agar well diffusion method showed that iron-scavenging metabolites produced by *N. mangyaensis* NH1 had a strong inhibitory activity towards mycelial growth in all tested fungal strains. The inhibition zone reached above 40.0 mm for *R. solani* MFP 936011 and *F. oxysporum* PR 57; the minimal and maximal zones being observed for *C. coccodes* MF 16-014 and *Alternaria* sp., respectively (Table 6, see Supplementary Fig. S10). The inhibition zone of *R. solani* MFP 936011 represented a viscous mucus layer in comparison to the dried inhibition zones of other fungi. For all fungi tested, the duration of antifungal activity of the metabolites varied. Stability of antifungal activity of metabolites *N. mangyaensis* NH1 lasted for 14–21 days for *R. solani* MFP 936011, *F. oxysporum* PR 57 and *C. coccodes* MF 16-014 and 5 days for *Alternaria* sp.. Difenoconazole was used as a positive control.

**Table 6:** Antifungal activity of metabolites produced by *N. mangyaensis* NH1 under Fe-limited conditions against phytopathogenic fungi.

Fungal strains	Diameter clear zone (mm)*	
	Metabolites of *N. mangyaensis* NH1	Difenoconazole^§^
*Colletotrichum coccodes* MF 16-014	41.7 ± 7.6	41.6 ± 4.7
*Rhizoctonia solani* MFP 936011	43.7 ± 7.1	NIZ
*Fusarium oxysporum* PR 57	50.5 ± 5.2	NIZ
*Alternaria* sp.	62.7 ± 14.2	NIZ

* All experiments were performed in triplicates.

^§^A systematic triazole fungicide. The concentration of difenoconazole was 1.2 mg/mL.

*NIZ* no inhibition zone.

#### Phytotoxic activity

The study explored the impact of metabolites produced by *N. mangyaensis* NH1, under conditions of iron-deficiency, on the growth of *Arabidopsis thaliana* plant seedlings. The results demonstrated that iron-scavenging metabolites of *N. mangyaensis* NH1 did not impact the growth of *Arabidopsis* seedlings compared to the control groups (untreated and treated with DMSO) (see Supplementary Figs. S11–13). Metabolites produced by *N. mangyaensis* NH1 in the presence of iron chloride III did not hinder the growth and development of plants either. We noted that the color of plants treated with metabolites from *N. mangyaensis* NH1 was less intense compared to untreated plants. *Arabidopsis* seedlings were additionally exposed to *N. mangyaensis* NH1 cells (2.8 ± 0.53 × 10*11 CFU/mL) to evaluate phytopathogenic activity. The findings suggest that *N. mangyaensis* NH1 did not impede the growth and development of *Arabidopsis* seedlings.

## Discussion

Endolithic systems are associated with environments permanently or seasonally exposed to various abiotic stresses, including desiccation/rehydration, temperature fluctuations and oligotrophy^40^. Despite severe environmental constraints, the microbial colonization of these systems by endoliths has significant evolutionary and ecological implications. Endolithic microbial communities are involved in the process of soil weathering, whereby they secrete metabolites into the soil surrounding minerals or rocks. Additionally, they play a role in the formation of soil aggregates and maintaining their structural stability^41^. Actinobacteria originating from endolithic environments have been described so far, including abundant genus *Streptomyces* and several rare genera *Actinomadura, Amycolatopsis, Nocardiopsis, Nonomuraea, Saccharopolyspora* and *Saccharothrix*^42^. It is well-known that *Actinobacteria* exhibit greater tolerance to extreme environments, such as arid, weathered or degraded soils. They have been found to bolster crop production under multiple stress conditions^43^.

Information on members of the genus *Nocardia* living in endolithic ecosystems is relatively limited. The cultivable *Nocardia* species isolated from stone ruins sampling in arid and semiarid climates in Tunisia were identified^44^. The adaptation strategy of actinobacteria to nutrient limitation and rock colonization was analyzed by studying the production of siderophores, hydrolytic enzymes, promoting plant growth and antimicrobial activity. Thus, endolithic habitats are a beneficial pool of potential sources for discovering unique *Nocardia* species that produce bioactive molecules including novel enzymes and secondary metabolites. This highlights the potential benefits of studying endolithic habitats as a rich resource for finding new species.

A significant number of biosynthetic gene clusters (BGCs) containing NRPS, PK or hybrid NRPS/PK enzymes encoding natural product production were found in *Nocardia* genomes^12^. A wide variety of gene clusters responsible for antibiotic synthesis by *Nocardia* species were demonstrated^6,12^. Despite this, a bioinformatic analysis revealed a highly conserved biosynthetic pathway leading to the formation of the siderophore nocobactin NA in both pathogenic and non-pathogenic *Nocardia* spp. Our findings reveal that the genome of *N. mangyaensis* NH1 comprises 5 gene clusters responsible for the production of siderophores. Additionally, nocobactine NA was confirmed by analysis via LC–MS/MS.

Non-targeted LC–MS/MS analysis showed that *N. mangyaensis* NH1 can synthesise membrane-bound mycobactins and non-membrane-bound carboxymycobactin-like compounds. Both types of siderophores were identified as siderophores that confer virulence to the genus *Mycobacterium*^27^. Considering that our strain was isolated from an endolithic environment, we hypothesize that these types of siderophores in *Nocardia* play significant physiological and ecological roles in enabling survival under extreme conditions. Beside the production of hydroxyphenyloxazoline/-oxazole type nocobactine and mycobactines, the strain *N. mangyaensis* NH1 was found to produce an amphiphilic peptide related to loihichelin-like siderophores. Loihichelins were initially identified and structurally characterized for the basalt weathering *Halomonas* strain LOB-5^29^. The authors concluded that siderophores play a direct role in both Fe^3^^+^ sequestering and Fe^2+^ oxidising during rock weathering. We have also discovered that *N. mangyaensis* NH1 synthesises siderophores similar to desferribactin, in combination with other compounds. Desferriferribactin is known as a biogenetic precursor for the biosynthesis of pyoverdins by *Pseudomonas fluorescens* ATCC 13525^45^. It is well known that members of the genus *Pseudomonas* are leaders among PGP bacteria, secreting diffusible siderophores that induce systemic plant resistance and plant pathogen resistance^46^. Thus, *N. mangyaensis* NH1 expresses a large number of genes that encode siderophores. The diversity of siderophores produced by strain NH1 contributes to the retention metals in bioavailable form in endolithic environments and indirectly stimulates the maintenance of soil fertility for plant survival.

The siderophore metabolome profile of *N. mangyaensis* NH1 exhibited a range of lipopeptide-like compounds. Lipopeptides are a type of biosurfactant produced by many bacteria^47^. The unique chemical composition of a peptide core and lipid tail plays a vital role in the ecological functions of lipopeptides, including protecting cells in extreme endolithic conditions, forming biofilms and chelating metals^48^. Lipopeptides produced by the bacterial genera *Bacillus* and *Pseudomonas* are widely studied for their antimicrobial and antifungal properties. *Bacillus* species produce bacillomycin, which has an impact on iron metabolism, root colonization, biofilm formation, and antifungal activity. Bacillomycin facilitates iron acquisition and its subsequent intracellular accumulation has a direct impact on biofilm formation through KinB-dependent induction in *B. velezensis* SQR9^34^. The antifungal activity of bacillomycins produced by *B. subtilis* FS 94-14 against the fungal plant pathogens *Ophiostoma ulmi, Verticillium dahliae*, *Ceratocystis fagacearum* and *Cryphonectria parasitica* has been demonstrated^49^. Similar to bacillomycins, bacillopeptins of *B. subtilis* FR-2 also well demonstrated antifungal and antimicrobial activities^33^.

We found that *N. mangyaensis* NH1 produces several cyclic peptides under Fe-deficient conditions. The metabolomic profile analysis revealed the presence of mycobacillin and mycosubtiline-like compounds. Isolated from *B. subtilis* species mycobacillin and mycosubtilin have the antifungal activity against plant pathogens^35,37^. Iron availability limitation was favorable for the synthesis of peptidolipins by *N. mangyaensis* NH1. For the first time, two peptidolipin NA derivatives were isolated from *N. arthritidis* IFM10035T co-cultivated with mouse macrophage cells^36^. Overcoming TRAIL-resistance and cytotoxic activities were found for *N. arthritidis* peptidolipins. Mycoplanecins-like moieties related to lipophilic cyclic peptide antibiotics with a specific mycobacterial activity were also annotated in a siderophore profile of *N. mangyaensis* NH1^38^. These findings demonstrate that strain NH1 produces a variety range of chemically distinct compounds alongside siderophores in conditions where iron is scarce. These compounds, which are related to lipopeptides and cyclic peptides, serve more than just as iron scavengers; they actively combat pathogenic bacteria and fungi, participate in the bioavailability and sequestering of metals, and are necessary for the stabilization of plant growth and development.

To determine functional properties of siderophores, mixture of metabolites produced by *N. mangyaensis* NH1 under iron deficiency was separated via preparative HPLC. CAS analysis of the purified compounds identified only four fractions belonging to the siderophore group. Screening for metal-chelating activity revealed that all CAS-positive fractions could form the Fe (III) complex. There is a high specificity for Fe(III) in the siderophore present in fraction 3. Only the siderophore from fraction 14 chelated all of the metals tested in this study. This indicates that the siderophore binding site from fraction 14 has a wide range of metal specificity. Siderophores from fractions 9–10 and 11 reacted with various metals, including copper (II), gallium (III), and iron. Therefore, we investigated the ability of siderophores produced by *N. mangyaensis* NH1 to chelate their natural ligand Fe (III) as well as other metals. Iron acquisition through microbial siderophores has been demonstrated in numerous studies^50^. Microbial siderophores promote metal sequestration in endolithic systems, accelerating soil mineral weathering, maintaining metal mobility and bioavailability, and enhancing plant growth^51^. In addition, chelation of metals by siderophores reduces their toxicity and promotes the production of microbial auxins^52^. We identified that *N. mangyaensis* NH1 is capable of producing IAA in a minimal medium using the same conditions as those for siderophore production. Along with the Fe (III)-binding activity of siderophores, it has also been demonstrated that IAA forms a complex with ferric iron, resulting in the subsequent formation of bioavailable ferrous iron^53^. These findings support the involvement of siderophores and a microbial-secreted phytohormone in the promotion of plant growth activity by actinobacterial strains^50^.

This study demonstrates a decrease in reactive oxygen species (ROS) by purified siderophores. The metal-binding specificity of *N. mangyaensis* NH1 siderophores was found to be correlated with their antioxidant activity. The siderophore with broad metal specificity did not exhibit ROS-protective properties, however, siderophores with a narrow range of metal binding specificity were found to possess antioxidant activity. The redox nature of siderophores, which mediates radical scavenging activity, has been referred to as one of their non-classical functions^14^. The production of siderophores by *Pseudomonas aeruginosa* protects the bacterium against ROS and facilitates the induction of bacterial cooperation under various environmental stressors^54^. Detoxification of ROS by catechol-type siderophores is well established in bacterial species belonging to the genera *Salmonella* and *Escherichia*^55,56^. This study demonstrated that the diverse siderophores produced by *N. mangyaensis* NH1 confer benefits that could shield plants against ROS, particularly in severely stressful endolithic, arid or weathered soil environments.

The mixture of compounds from siderophore metabolome of *N. mangyaensis* NH1 showed antifungal activity against the phytopathogenic fungi *F. oxysporum, R. solani, C. coccodes* and *Alternaria* sp. Fungi are one of the dominated members in the endolithic microbial communities^40^. The presence of phytopathogenic fungi decreases the plant fitness in extreme conditions. Several studies have demonstrated the efficacy of siderophore-producing PGP bacteria in controlling fungal phytopathogens, but there are few reports detailing the antifungal activity of purified siderophores. For instance, an acinetobactin-like siderophore secreted by the wheat rhizospheric strain *Acinetobacter calcoaceticus* HIRFA32 inhibited *F. oxysporum *in vitro^57^. Pyoverdine produced by *P. aeruginosa* JAS-25 isolated from the saprophytic soil was found to inhibit the spore germination of *F. oxysporum* f. sp. *ciceri*, *F. udum*, and *Aspergillus niger*^58^. Thus, we have shown the direct antifungal activity of metabolites secreted by *N. mangyaensis* NH1 under Fe-deficient conditions as a valuable skill in the interactions between plant-microbial communities and the management of plant diseases.

In addition to the participation of *N. mangyaensis* NH1 siderophores in ROS protection, metal-binding, and antifungal activities, the effects of metabolome compounds on a model plant *A. thaliana* have been demonstrated. Plant seedlings cultivated in the iron-free medium with metabolites produced by *N. mangyaensis* NH1 were found to develop without any visually negative effects. However, the color of plants varied between treated and untreated plants. We propose that the alteration in color to a pale green hue in treated plants of *A. thaliana* could be ascribed to the chlorophyll content or chlorophyll a/b ratio in the foliage influenced by the availability of iron ions for plants.

Indeed, the *N. mangyaensis* NH1 siderophores exhibited a suppression of phytopathogenic fungi while lacking phytotoxicity. There is considerable evidence that microbial siderophores help promote plant growth, suppress disease and facilitate iron uptake^59^. However, only a limited number of bacterial siderophores have been purified and analyzed in plant experiments. The siderophore rhizoferrin produced by *Rhizopus arrhizus* serves as a source of Fe for barley (*Hordeum vulgare* L.) and corn (*Zea mays* L.)^60^. A positive influence of pseudobactin excreted by *Pseudomonas putida* strain WCS358 has been shown to serve on plant iron nutrition and chlorophyll synthesis^61^. Therefore, we propose that the siderophores of *N. mangyaensis* NH1 can be regarded as metabolites that provide a physiologically relevant amount of iron to plants under iron deficiency conditions.

## Materials and methods

Genomic DNA from strain NH1 was extracted from a 96 h LA-grown culture using phenol–chloroform method. A paired-end DNA library was prepared using the NEBNextUltra II DNA Library Prep Kit (Illumina) and NEBNext Multiplex Oligos for Illumina (96 Unique Dual Index Primer Pairs) according to manufacturer guidelines. Whole-genome sequencing was carried out by NovaSeq6000 sequencing platform (Illumina) with 2 × 250 bp read length. The quality of raw sequence reads was evaluated by FastQC package (v0.11.3)^62^. The genome had an average 446.4X sequencing coverage. Overrepresented and low-quality sequences were removed using Trimmomatic (v0.36)^63^. The whole genome was assembled using SPAdes (v 3.12) software^64^. The genome assembly was evaluated using QUAST (v4.6.3) program^65^. Genome annotation was performed by RAST 2.0 server, Prokka (v1.14.6) annotation pipeline and the NCBI Prokaryotic Genomes Annotation Pipeline (PGAP) version 5.2 (see Supplementary Table S2)^66^.

### 16S rRNA gene analysis

Pairwise sequence similarities calculated using 16S rRNA genes available via the GGDC web server (http://ggdc.dsmz.de/) showed that strain NH1 clusters with the *N. mangyaensis* (see Supplementary Fig. S1).

The Average Nucleotide Identity (ANI) of the strain NH1 genome was analyzed using FastANI^67^.

Biosynthetic gene clusters were predicted to be present in the genome of strain NH1 by the antiSMASH software 7.0^68^.

### Indole-3-acetic acid (IAA) detection

The culture supernatant were mixed with methanol and centrifuged at 13,000 rpm, 30 min for complete protein precipitation. An aliquot of the resulting mixture was then used for LC–MS/MS analysis. Calibration curve of IAA (Acros) were constructed for 7.8, 15.6, 31.25, 62.5, 125, 250 and 500 ng/µL. The ABSciex QTRAP 6500 coupled with a Agilent 1290 Infinity (LC/MS/MS System), equipped with an electrospray ion source, and operated in the positive ion mode was used. A column Discovery HS C18 3 μm, 50 × 2.1 mm (Sigma-Aldrich, USA) with mobile phases A (5% acetonitrile and 0.1% formic acid in water) and B (5% water and 0.1% formic acid in acetonitrile) was used. The column temperature was set at 40 °C. Chromatographic separation was achieved by gradient elution at a flow rate of 0.4 mL/min. The gradient program was as follows: 0–1.0 min from 0 to 2% B; 1.0–6.0 min from 2 to 95% B; 6.0–7.0 min 95% B; 7.0–8.0 min from 95 to 2% B; and 8.0–9.0 min 2% column re-equilibration. The injection volume of 10 µL was used.

MRM (multiple reactions monitoring) transitions and mass spectrometry parameter optimization were performed by direct infusion using standard solution of IAA. The MRM transitions were: m/z 176.0 → 77.0, m/z 176.0 → 103.2, m/z 176.0 → 130.0. The daughter ion with m/z 130.1 used for quantification. The source parameters used in the study were: temperature—500 °C, capillary voltage—5200 V, curtain gas pressure—35 psi, nebulizer gas pressure—55 psi, auxiliary gas pressure—55 psi. The mass spectrometric data were analyzed using a MultiQuant 3.0.2 software (AB Sciex) (see Supplementary Fig. S3).

Isolation of siderophores from the culture supernatant. A preliminary CAS agar plate assay was conducted to assess the production of siderophores by strain NH1 (see Supplementary Fig. S5)^69^. *N. mangyaensis* NH1 was grown for 72 h in M9 medium under iron-limited conditions. Catecholate- and hydroxamate-type siderophores in solution were detected using the Arnow and Atkin assays, respectively^70,71^. All experiments were performed in triplicates.

After extraction of the culture supernatant with XAD16 resin, the adsorbed compounds were eluted with methanol and subjected to Orbitrap LC–MS/MS. Untargeted LC–MS/MS analysis was performed as described previously^72^. The data obtained from HPLC–MS/MS analysis were converted to .mzML format using the MsConvert software^73^. Data processing was performed by using the software MZMine 2^74^. The data processing workflow included: (1) mass detection; (2) ADAP chromatogram builder^48^; (3) smoothing; (4) deconvolution; (5) deisotoping; (6) alignment; (7) gap filling. The LC–MS/MS processed and converted files (.mzML) were uploaded to the global natural products social molecular networking (GNPS) platform (https://gnps.ucsd.edu/ProteoSAFe/static/gnps-splash.jsp) with Dereplicator+, MolDiscovery and VarQuest (Analog Search) computational tools^65,75,76^. Network parameters were default. The networks were visualized using the Cytoscape network visualization tool/software 3.10.1^77^.

### Preparative HPLC (prep-HPLC)

The vacuum-dried metabolites, which were dissolved in methanol and directly injected into the column, were purified using the Puri Flash 450 Interchim HPLC instrument.. A C18-reversed phase silica gel column (15 µm, 40 g) was utilized in gradient elution mode (0 min water/acetonitrile = 100–0 to 60 min water/acetonitrile 0–100) at a flow rate of 5 ml/min. Absorbance at 256 and 340 nm was monitored during this procedure, resulting in the separation of the CAS-positive fractions (tR = 3.0, 9.0–10.0, 11.0 and 14.0 min).

### Antioxidant activity

Hydrogen peroxide was detected in the presence of the CAS-positive fractions (2 µg/mL HPLC-purified CAS-positive fractions) using the Amplex™ Red Hydrogen Peroxide/Peroxidase Assay Kit, following the manufacturer’s protocol (Invitrogen). A positive control of 10 µM hydrogen peroxide and a negative control of water were employed.

### Metal-binding activity

All metals, CoCl_2_ × 6H_2_O, AlCl_3_, NiSO_4_ × 7H_2_O, FeCl_3_ × 6H_2_O, ZnSO_4_, MnSO_4_, CuSO_4_ and GaBr_3_ were prepared in 50 mM concentration in 0.5N HCl. The final concentration of the tested metals was 100 µM. For each metal tested, 2 µg/mL of the HPLC-purified CAS-positive fraction was added. The siderophores and metals were mixed and left to incubate at room temperature overnight. The absorbance spectra were observed within the wavelength range of 230–700 nm.

### Antifungal activity

The spores of *Fusarium oxysporum* PR57, *Colletotrichum coccodes* MF 16-014, *Rhizoctonia solani* MFP 936011 and *Alternaria* sp. (1 × 10^8^ CFU/mL) were spread on the Czapek's agar (CZA) plates. 10 µL of the siderophore mixture (1.2 mg/ml) was added to the well in the centre of the plate. A control plate was inoculated with fungal spores alone, while another plate was inoculated with 10 µL of methanol as the negative control. The positive control was a mixture of metabolites (1.2 mg/mL) secreted by *N. mangyaensis* NH1 in the presence of FeCl_3_. The plates were incubated for 3–5 days at 30 °C until fungal mycelia fully covered the agar surface in the control plate. The diameter of the inhibition zone was measured in mm. A solution of commercial difenoconazole (250 g/L, Raek, KE^®^, Avgust Crop Protection, Russia) was used as a positive control (Line 504–505). The 0.312 mg/mL working solution has been tested as recommended for agricultural practice (State Catalogue of Pesticides and Agrochemicals, 2023) for the treatment of tomatoes and potatoes against phytopathogens^78^ and 1.2 mg/mL, which is equivalent to a tested mixture of metabolites secreted by *N. mangyaensis* NH1. All experiments were performed in triplicates.

### Plant treatment assay

The influence of metabolites produced by *N. mangyaensis* NH1 under iron-deficiency conditions on plants was performed using *Arabidopsis thaliana* plants. *A. thaliana* (L.) Heynh. wild-type accession Col-0 (CS6673) seeds were purchased from the Arabidopsis Biological Resource Center (ABRC, https://abrc.osu.edu/). Seeds were surface sterilized using 50% bleach and plated on ½ strength Murashige and Skoog medium with 2% sucrose and 0.7% agar. Seeds were incubated for 7 days in a growth chamber under a 16-h light/8-h dark cycle at 20 °C. Cotyledon-formed seedlings were transferred to new plates with the same medium and grown for 7 days under the standard conditions before reaching 6–7 true leaves.

*Nocardia mangyaensis* NH1 metabolites produced under iron-deficiency and iron-enriched conditions were dissolved in DMSO in concentrations of 1.2 mg/mL and 0.6 mg/mL. Then 5 uL of metabolites were applied on a plant under the leaf rosette. DMSO-treated and non-treated plants were used as a negative control. Plates with plants were incubated for 10 days under standard conditions. Plant phenotypes (leaf rosette size, leaf color (chlorophyll content), root branching) were monitored every day and characterized on the 10th day of incubation.

### Plant infection assay

Plant infection assay was performed as it was described previously^72^. *N. mangyaensis* NH 1 strain was cultured in LB medium at 30 °C on an orbital shaker with 250 rpm agitation for 96 h. All treatments of *A. thaliana* seedlings by NH1 strain were performed in triplicates.

All methods were carried out in accordance with relevant guidelines and regulations. All necessary permissions for planting and investigating this cultivar were obtained from South China Agricultural University, and the collection and research of this cultivar have complied with the Convention on the Trade in Endangered Species of Wild Fauna and Flora.

## Conclusions

We conducted a detailed study of a 'rare' endolithic bacterium isolated from hydromagnesite and identified as *Nocardia mangyaensis* NH1. Genomic analysis of strain NH1 showed a highly diverse biosynthetic capacity for the production of natural products. *N. mangyaensis* NH1 demonstrates the secretion of siderophores and the synthesis of indole acetic acid. Here, we present a detailed annotation of the siderophore metabolome of the bacterium. A significant amount of metabolites belonging to the categories of siderophores, lipopeptides and cyclic peptides produced by *N. mangyaensis* NH1 during cultivation under Fe-limited conditions was discovered. Metal-binding screening of *N. mangyaensis* NH1 siderophores revealed their high, narrow and broad specificity to metals. Siderophores with high and specific metal chelating activity have antioxidant properties. We have shown that the siderophore metabolites of *N. mangyaensis* NH1 exhibit antifungal activity while having no deleterious effects on the model plant *A. thaliana*. The study concludes that by inoculating soil, particularly in degraded and arid regions, with the endolithic bacterium *N. mangyaensis* NH1, the agronomic effectiveness can be improved through enhanced mineral availability. Furthermore, soil fertility can be increased and crops can be protected against phytopathogens and oxidative stress.

### Supplementary Information

Supplementary Information.

## Data Availability

This whole-genome shotgun project has been deposited at DDBJ/ENA/GenBank under the accession JAUMIP000000000. The data sets exhibited in this research can be located within digital repositories available online (GNPS). The metadata can be found on https://massive.ucsd.edu/, MSV000093024.
