# Supplementary material for: Genomic insights and anti-phytopathogenic potential of siderophore metabolome of endolithic Nocardia mangyaensis NH1
Source: Sci Rep. 2024 Mar 7;14:5676. doi: 10.1038/s41598-024-54095-9 (PMC10920908; doi:10.1038/s41598-024-54095-9)
Supplement: Supplementary file 1 — Supplementary Information. [file 41598_2024_54095_MOESM1_ESM.docx]

**Supplemental Information**

Genomic insights and anti-phytopathogenic potential of siderophore metabolome of endolithic *Nocardia mangyaensis* NH1

Irina V. Khilyas*^1^, Maria I. Markelova^2^, Lia R. Valeeva^1^, Tatiana M. Ivoilova^1^, Elena I. Shagimardanova^3^, Alexander V. Laikov^2^, Anna A. Elistratova^1^, Ekaterina S. Berkutova^1^, Guenter Lochnit^4^ and Margarita R. Sharipova^1^

^1^ Department of Microbiology, Institute of Fundamental Medicine and Biology, Kazan (Volga Region) Federal University, Kazan, Russian Federation;

^2^ Laboratory of Omics Technologies, Institute Fundamental Medicine and Biology, Kazan (Volga Region) Federal University;

^3^ Laboratory of Extreme Biology, Institute of Fundamental Medicine and Biology, Kazan (Volga Region) Federal University, Kazan, Russian Federation

^4^ Protein Analytics, Institute of Biochemistry, Faculty of Medicine, Justus Liebig University Giessen, Giessen, Germany

***** Correspondence: Irina V. Khilyas, irina.khilyas@gmail.com

|  |
| --- |

Supplemental Figures S1-S13

Supplemental Table S1-S2

**
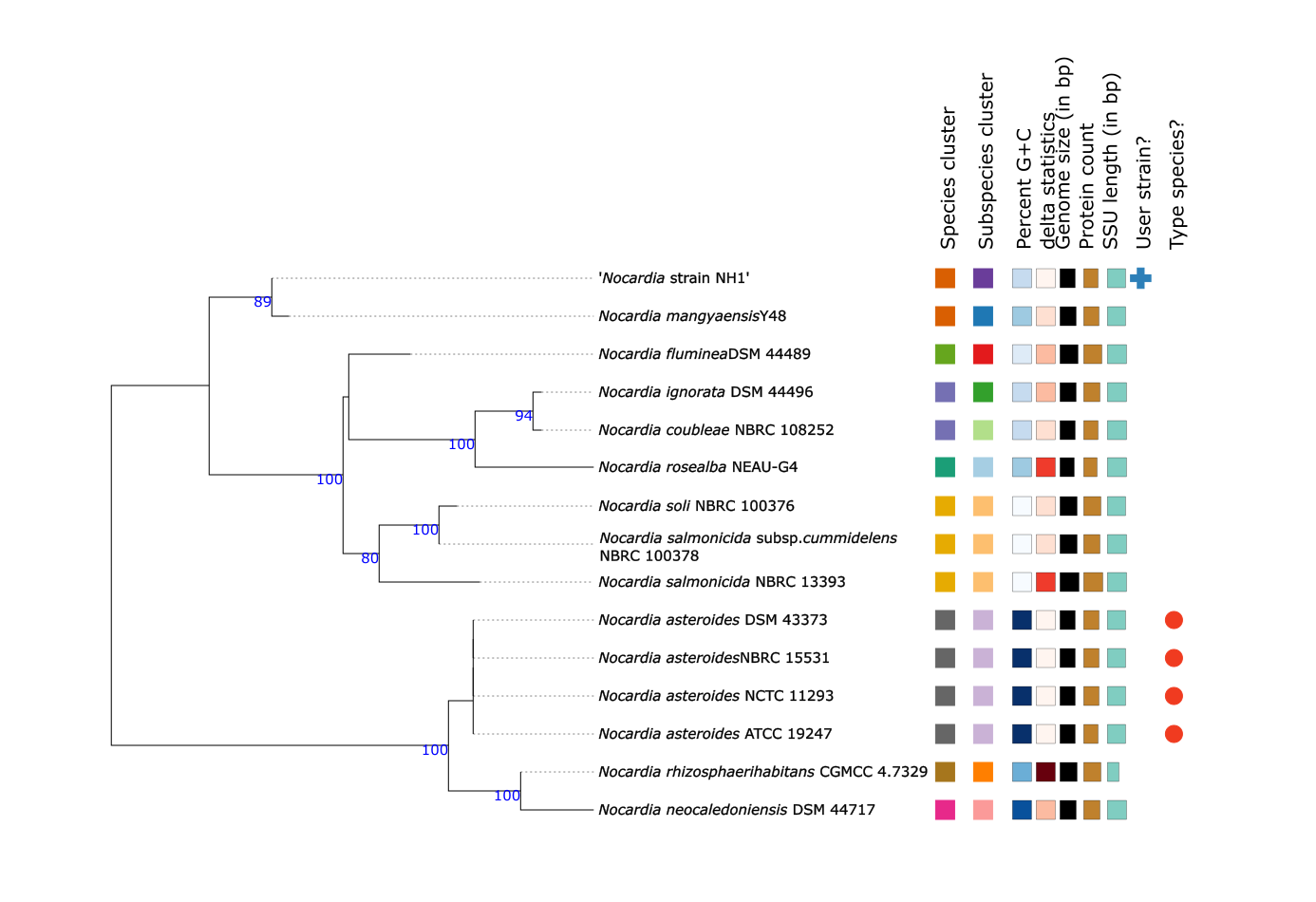
**

**Figure S1.** Phylogenetic tree based on neighbour-joining analysis of the 16S rRNA gene sequence similarities of the *Nocardia* strains. The branch lengths are scaled in terms of GBDP distance formula d5. The numbers above branches are GBDP pseudo-bootstrap support values > 60 % from 100 replications, with an average branch support of 75.0 %. The tree was rooted at the midpoint.

**Table S1**. Pairwise comparisons of *Nocardia mangyaensis* NH1 genomes vs. *Nocardia* strain genomes.

| Type Strain Genome Server | | | | | | | | |
| --- | --- | --- | --- | --- | --- | --- | --- | --- |
| **Query strain** | **Subject strain** | **dDDH (d0, in %)** | **C.I. (d0, in %)** | **dDDH (d4, in %)** | **C.I. (d4, in %)** | **dDDH (d6, in %)** | **C.I. (d6, in %)** | **G+C content difference (in %)** |
| 'Nocardia strain NH1' | Nocardia mangyaensis Y48 | 73,9 | [69.9 - 77.5] | 70,9 | [67.9 - 73.7] | 75,9 | [72.5 - 79.1] | 0,09 |
| 'Nocardia strain NH1' | Nocardia rhizosphaerihabitans CGMCC 4.7329 | 39 | [35.7 - 42.5] | 30,1 | [27.8 - 32.7] | 36,1 | [33.1 - 39.1] | 0,42 |
| 'Nocardia strain NH1' | Nocardia asteroides NBRC 15531 | 39,5 | [36.2 - 43.0] | 29,3 | [26.9 - 31.8] | 36,2 | [33.2 - 39.2] | 1,92 |
| 'Nocardia strain NH1' | Nocardia asteroides ATCC 19247 | 39,9 | [36.5 - 43.4] | 29,3 | [26.9 - 31.8] | 36,5 | [33.5 - 39.5] | 2,03 |
| 'Nocardia strain NH1' | Nocardia asteroides NCTC 11293 | 39,6 | [36.3 - 43.1] | 29,3 | [26.9 - 31.8] | 36,3 | [33.3 - 39.3] | 1,88 |
| 'Nocardia strain NH1' | Nocardia asteroides DSM 43373 | 39,5 | [36.1 - 42.9] | 29,2 | [26.9 - 31.7] | 36,1 | [33.2 - 39.2] | 1,93 |
| 'Nocardia strain NH1' | Nocardia neocaledoniensis DSM 44717 | 38,3 | [34.9 - 41.8] | 28,8 | [26.4 - 31.3] | 35,1 | [32.1 - 38.1] | 1,67 |
| 'Nocardia strain NH1' | Nocardia salmonicida subsp. cummidelens NBRC 100378 | 39,8 | [36.4 - 43.2] | 28,4 | [26.0 - 30.8] | 36,1 | [33.1 - 39.1] | 0,93 |
| 'Nocardia strain NH1' | Nocardia salmonicida NBRC 13393 | 37,4 | [34.0 - 40.9] | 28,3 | [25.9 - 30.8] | 34,2 | [31.3 - 37.3] | 0,97 |
| 'Nocardia strain NH1' | Nocardia fluminea DSM 44489 | 36,7 | [33.3 - 40.2] | 28,3 | [25.9 - 30.8] | 33,8 | [30.8 - 36.8] | 0,51 |
| 'Nocardia strain NH1' | Nocardia soli NBRC 100376 | 39,7 | [36.4 - 43.2] | 28,3 | [25.9 - 30.8] | 36 | [33.1 - 39.1] | 0,95 |
| 'Nocardia strain NH1' | Nocardia coubleae NBRC 108252 | 35,3 | [31.9 - 38.8] | 27,3 | [24.9 - 29.8] | 32,4 | [29.4 - 35.5] | 0,05 |
| 'Nocardia strain NH1' | Nocardia ignorata DSM 44496 | 33,5 | [30.2 - 37.1] | 27,1 | [24.8 - 29.6] | 31 | [28.1 - 34.1] | 0,28 |
| 'Nocardia strain NH1' | Nocardia rosealba NEAU-G4 | 37,4 | [34.1 - 40.9] | 27,1 | [24.7 - 29.6] | 33,9 | [31.0 - 37.0] | 0,03 |

**Table S2.** Comparison of *N.* *mangyaensis* NH1 genomic annotations using PGAP^*^ and RAST^**^.

| Genomic feature | PGAP | RAST |
| --- | --- | --- |
| Total number of genes | 6279 | 6443 |
| Protein coding genes | 6123 | 6384 |
| Number of RNAs | 66 | 59 |
| Contigs | 206 | 211 |
| N50 | 283 780 bp | 194,708 bp |
| GC% | 68 | 68 |

^*^  PGAP - NCBI Prokaryotic Genomes Annotation Pipeline

^**^ RAST - Rapid Annotation using Subsystem Technology


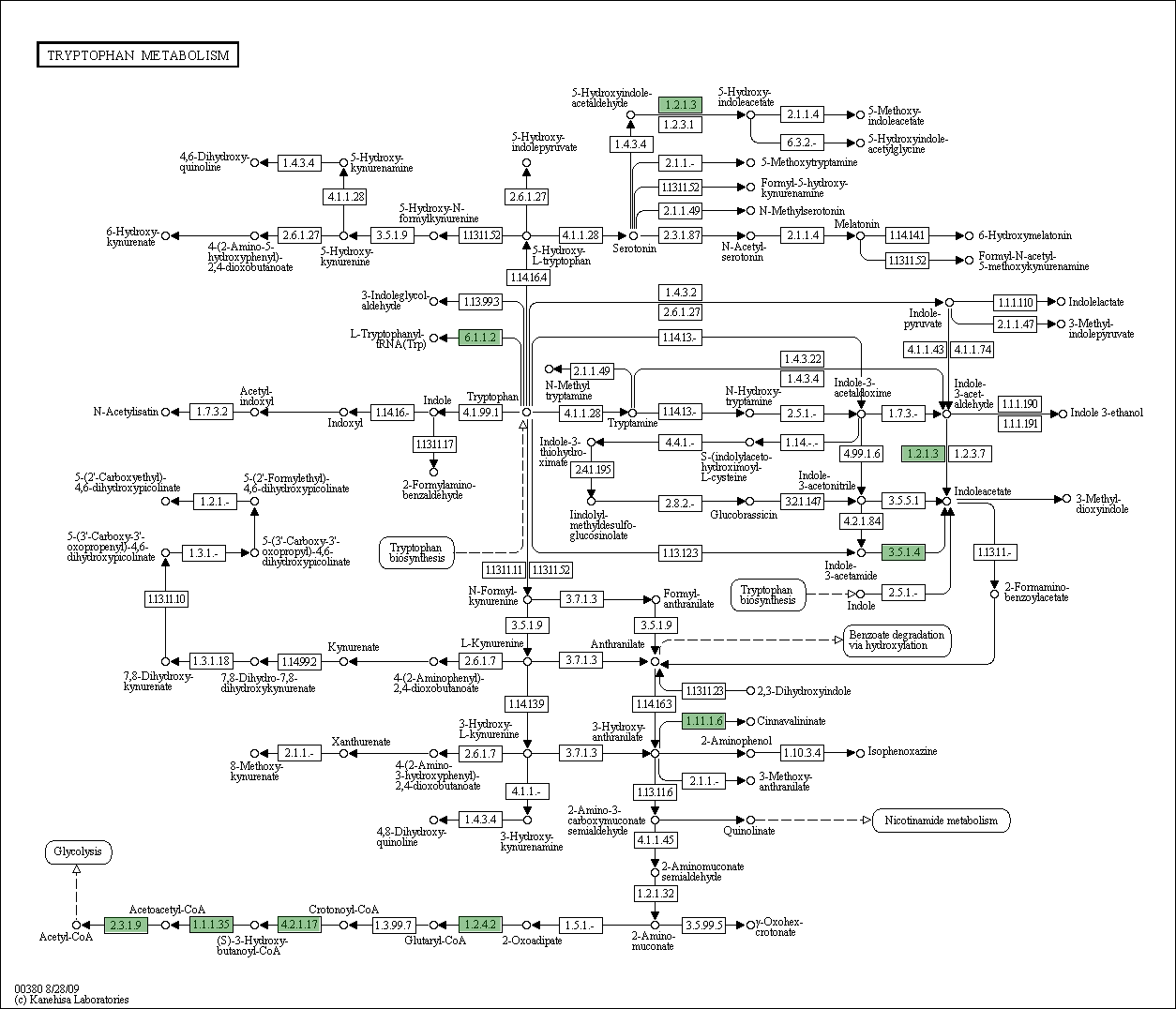


**Figure S2.** KEGG pathway mapping of the tryptophan metabolism (https://www.kegg.jp/kegg/kegg1.html). Green markers are genes related to the IAA synthesis pathway identified in the *N. mangyaensis* NH1 genome.

GNPS JOB LINKS

1. Dereplicator:

<https://gnps.ucsd.edu/ProteoSAFe/status.jsp?task=012d433df31e4b95b8f7e8ac8fdd2d64>

1. Dereplicator plus:

https://gnps.ucsd.edu/ProteoSAFe/status.jsp?task=63dbbd1401bc4336bc7a8e2acf4e9d93

1. Moldiscovery:

<https://gnps.ucsd.edu/ProteoSAFe/status.jsp?task=b8b97e2590184198ac8200bf1ea2967d>

1. Metabolomics-snets-v2

<https://gnps.ucsd.edu/ProteoSAFe/status.jsp?task=5c69183f3c6f446295b2c8e715505ae4>
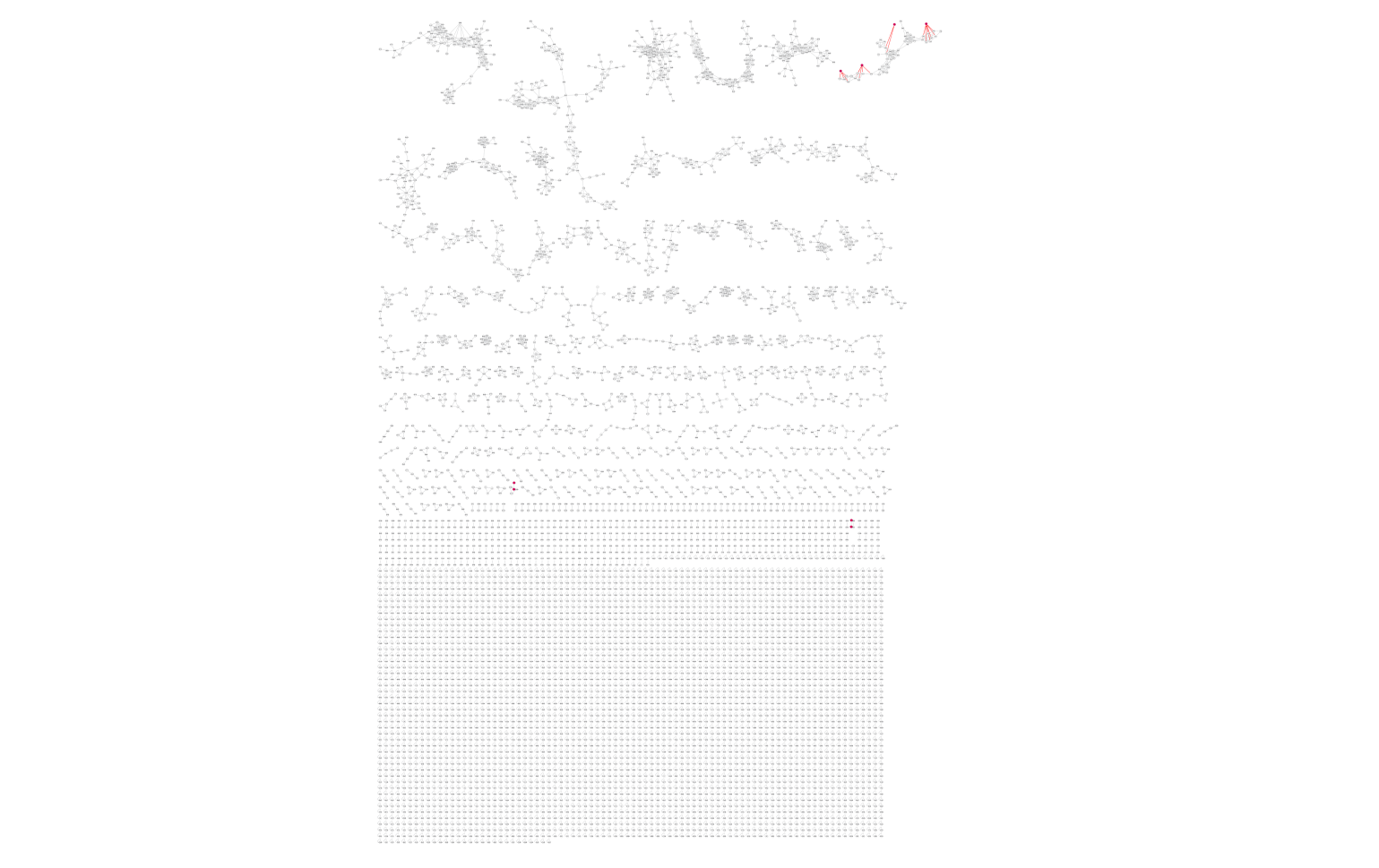

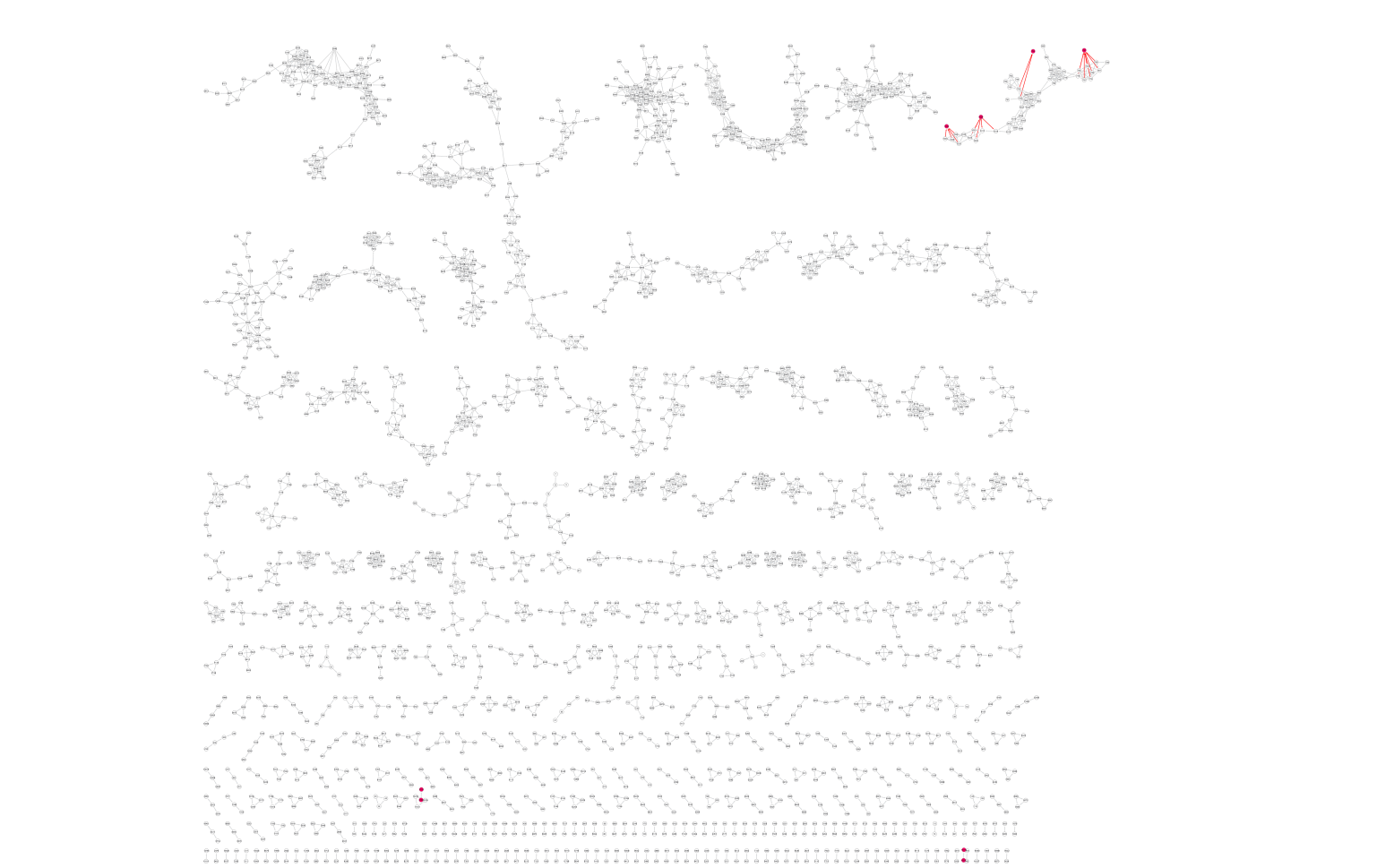


**Figure S3**. Molecular network of the ions detected in the culture supernatant of *N. mangyaensis* NH1 grown under iron-limited conditions. Grey nodes represent all the ions detected in the culture supernatant, red nodes represent the siderophore- and lipopeptide-like compounds: (A) a whole spectrum of detected ions; (B) the magnified area of the siderophore- and lipopeptide-like compounds.

B

A

**
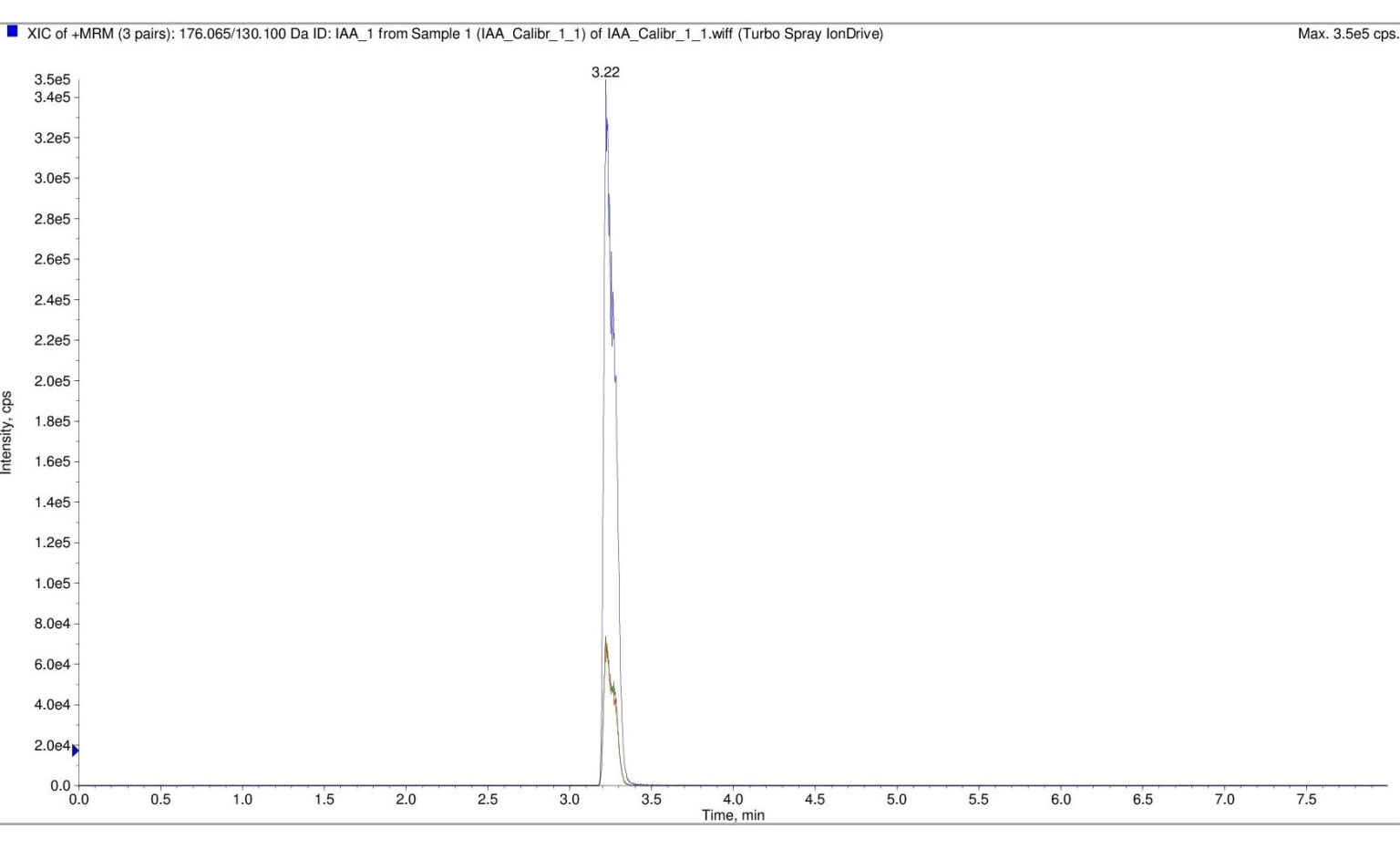
**

A

B

**
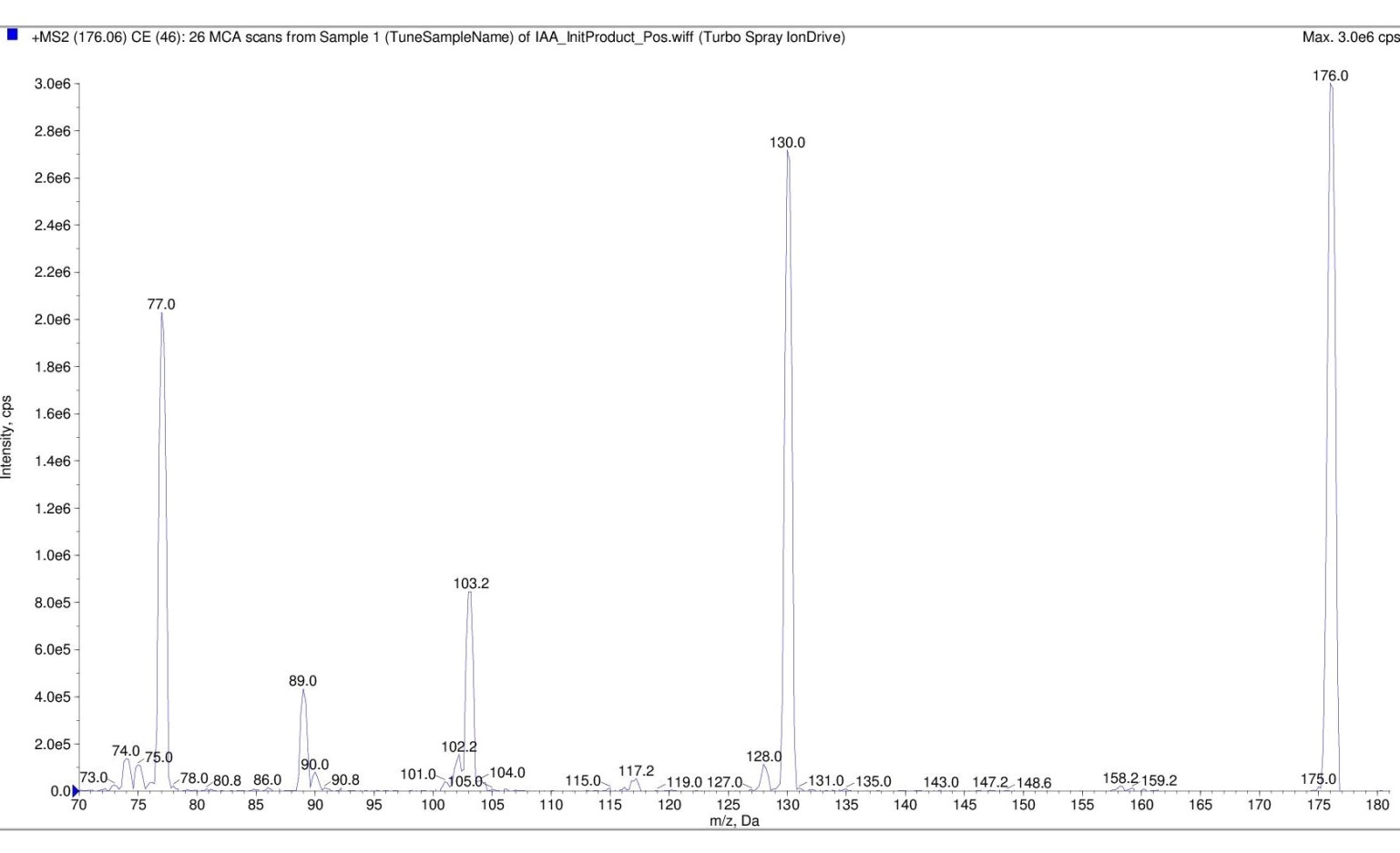
**

**
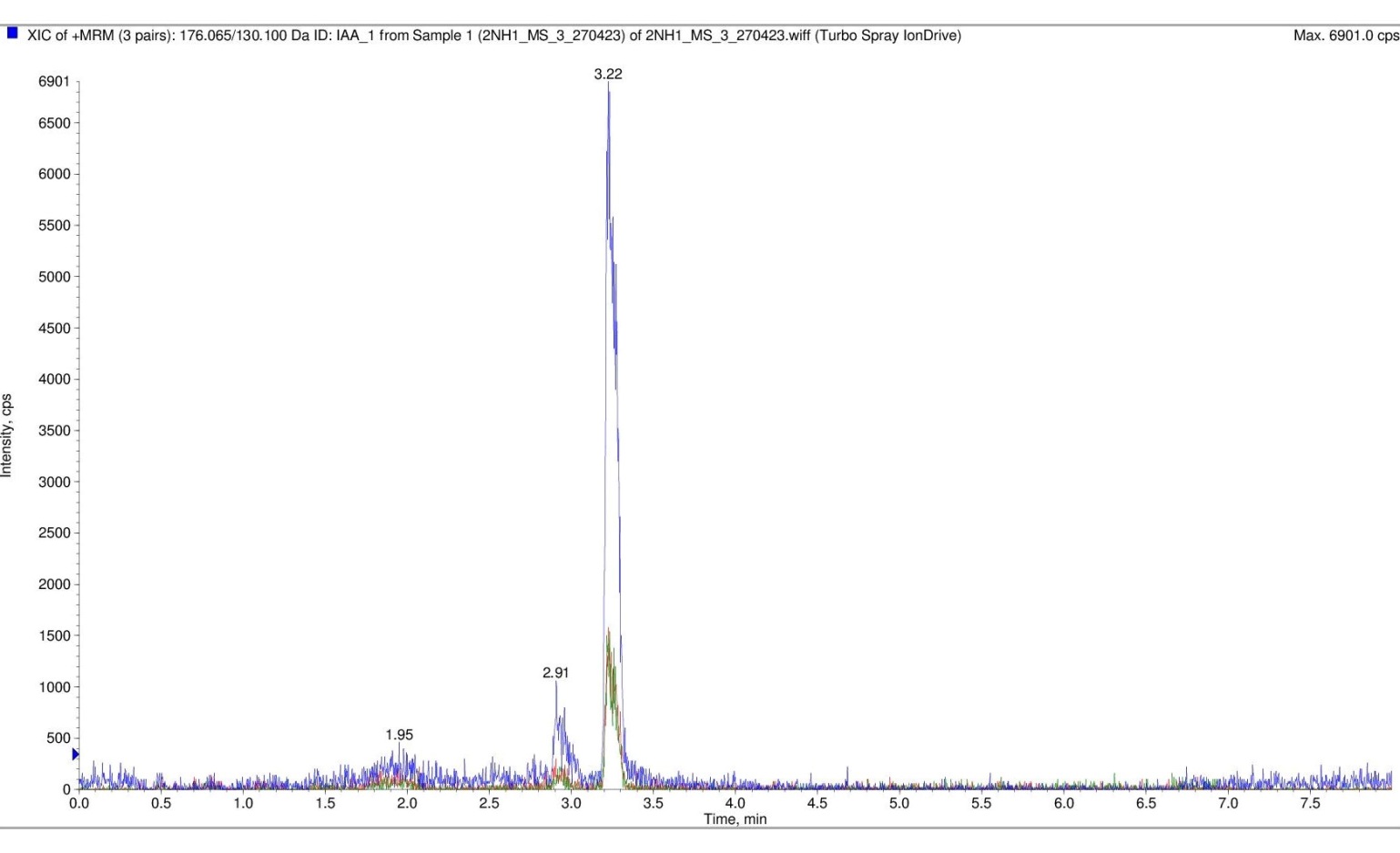
**

C

**Figure S4.** MRM chromatogram (176.0→130.0 m/z, 176.0→103.2 m/z and 176.0→77.0 m/z) of standard sample indole-3-acetic acid (IAA) (A) and its mass spectrum (B). MRM chromatogram of IAA in the culture liquid of *N. mangyaensis* NH1 grown for 72 h in M9 medium under iron-limited conditions (C). All measurements were carried out in triplicate.

.


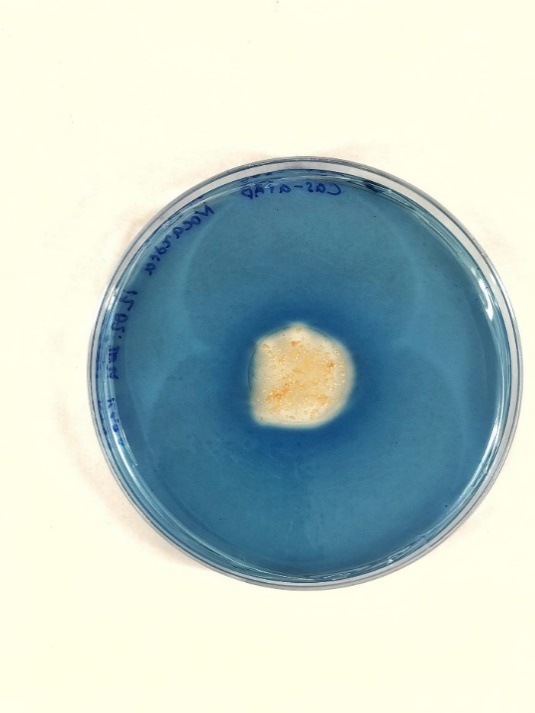


**Figure S5**. CAS agar assay for the production of siderophores by *Nocardia mangyaensis* NH1.

*
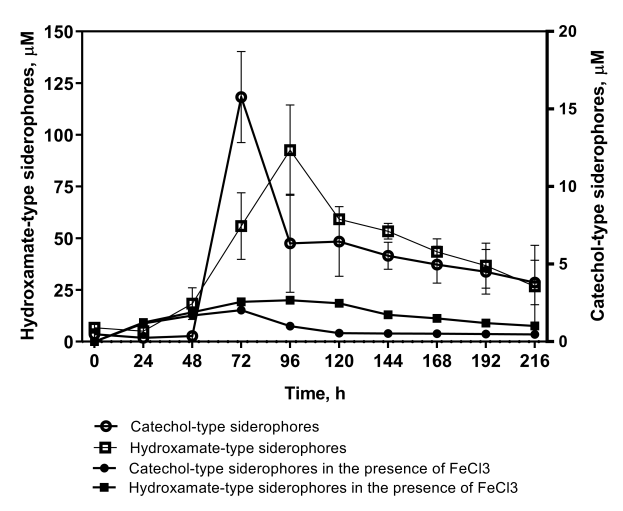
*

**Figure S6**. Secretion of siderophores by N. mangyaensis NH1 in the liqued M9 medium in the presence/absence of iron chloride III. Error bars represent the standard deviation of triplicate experiments.

**Figure S7**. HPLC chromatogram of a mixture of metabolites produced by *N. mangyaensis* NH1 grown under Fe-deficient conditions (black line) and metabolites produced by *N. mangyaensis* NH1 grown in the presence of FeCl_3_ (red line).

**
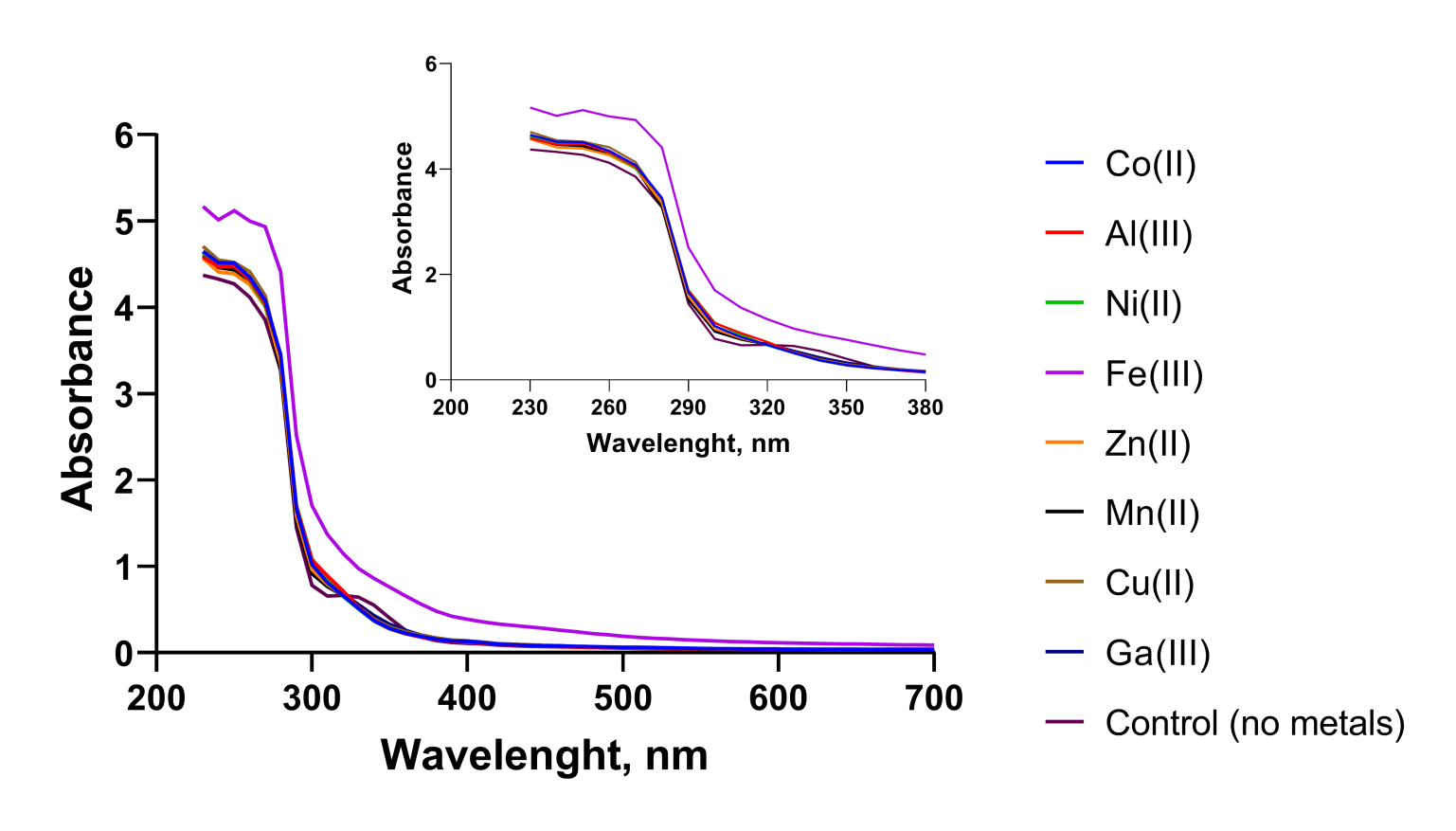
**

**B**

A

**
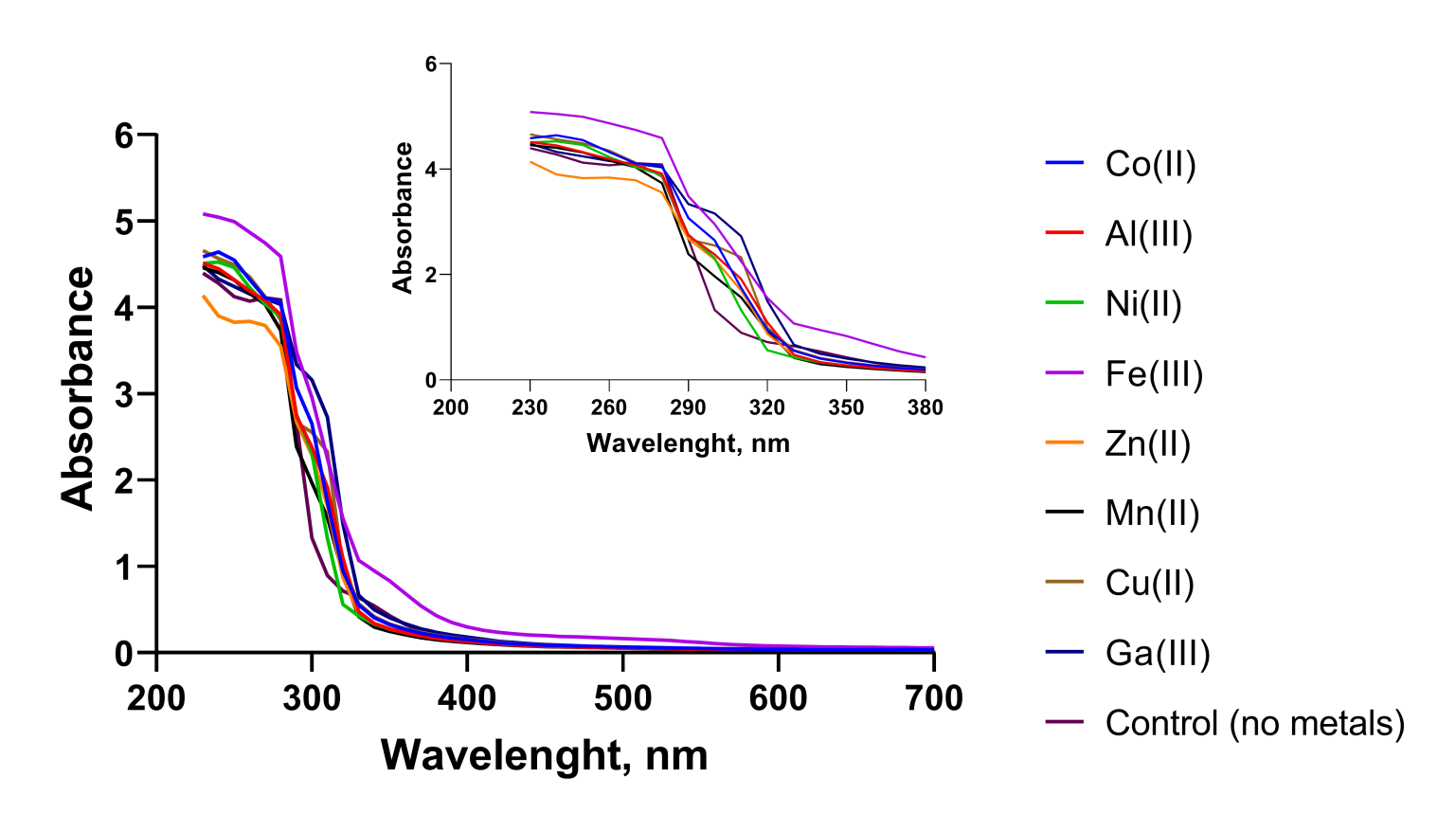
**

**
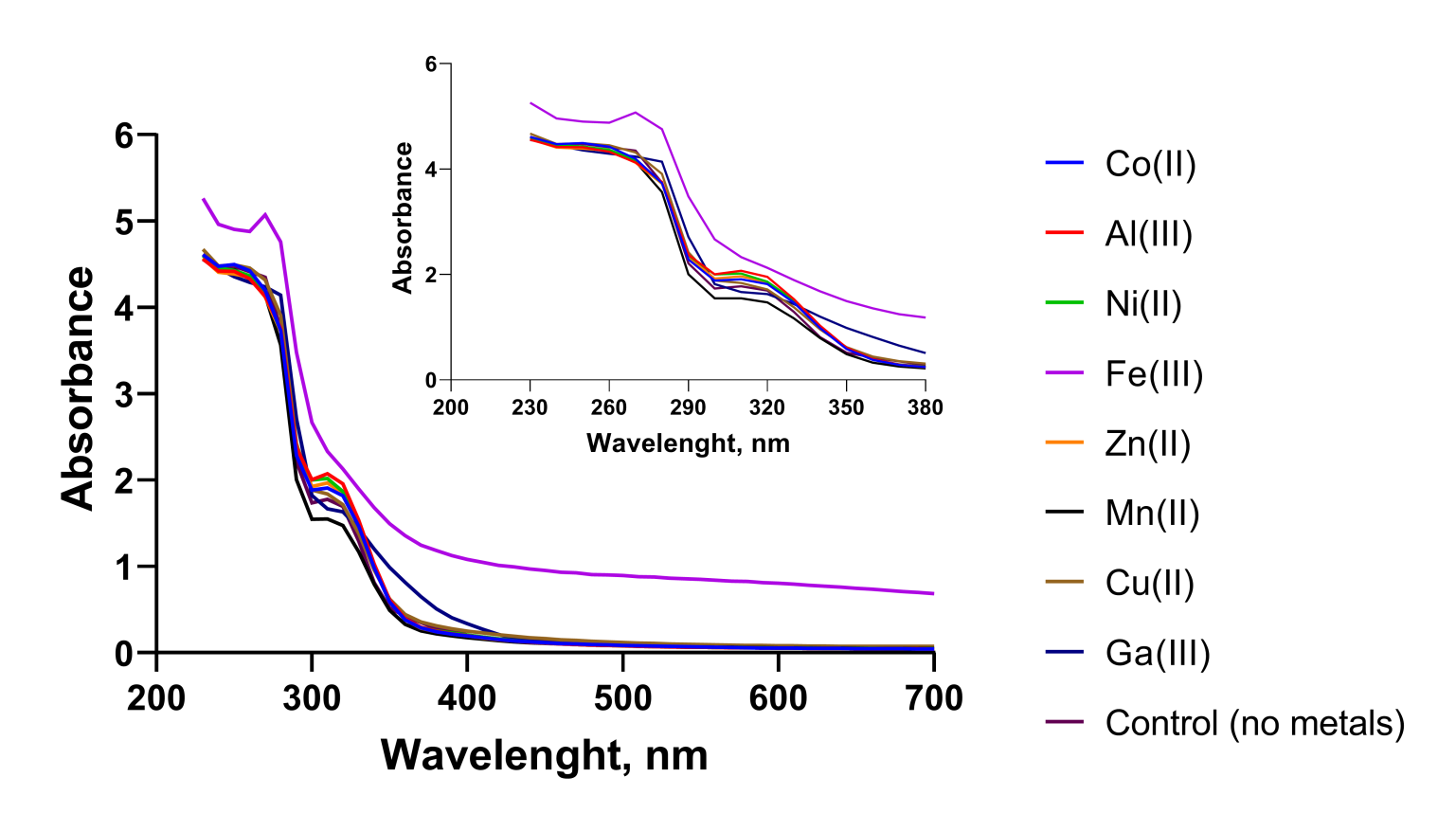
**

C

D

**
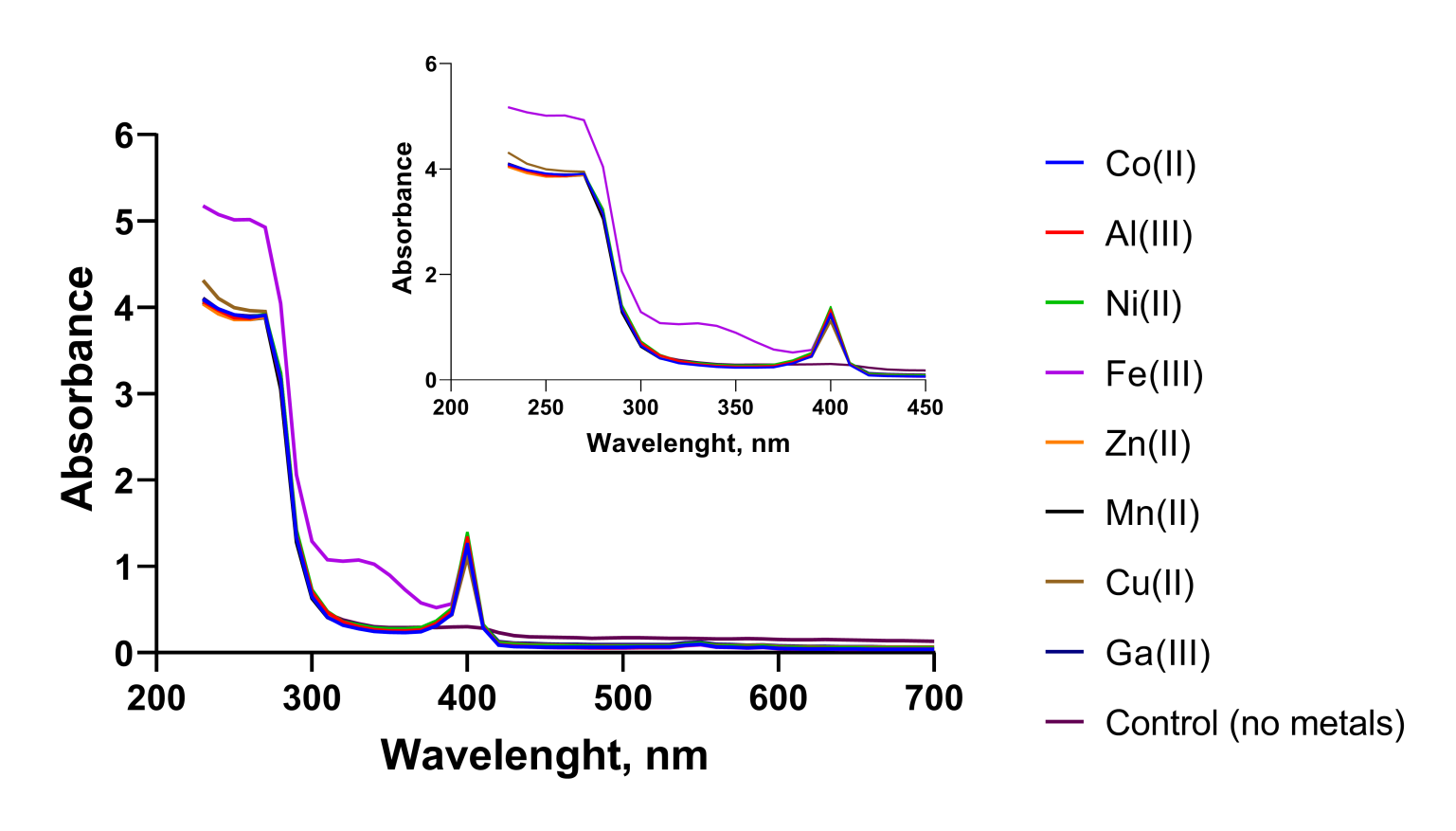
**

**Figure S8**. UV/vis absorption spectra of (A), fraction 3, (B), fraction 9-10, (C), fraction 11 and (D), fraction 14 at a concentration of 2 µg/mL incubated with 50 mM of metal salts for 22h at room temperature. The control contained only water.

*
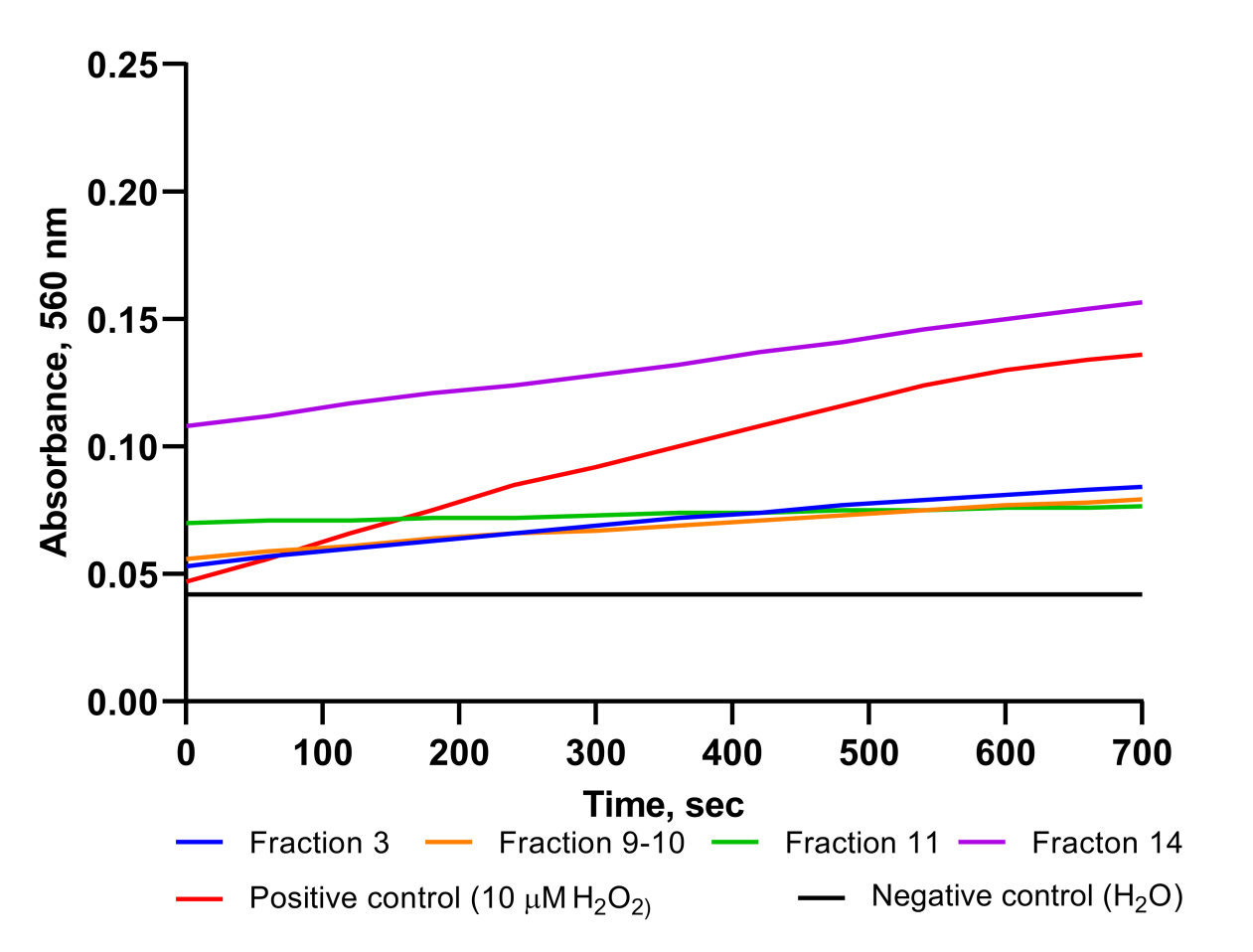
*

**Figure S9.** Amplex Red/HRP enzymatic coupled assay of HPLC-purified fractions of siderophores produced by *N. mangyaensis* NH1. The positive control was 10 µM H2O2 and the negative control was water without H_2_O_2_.

**
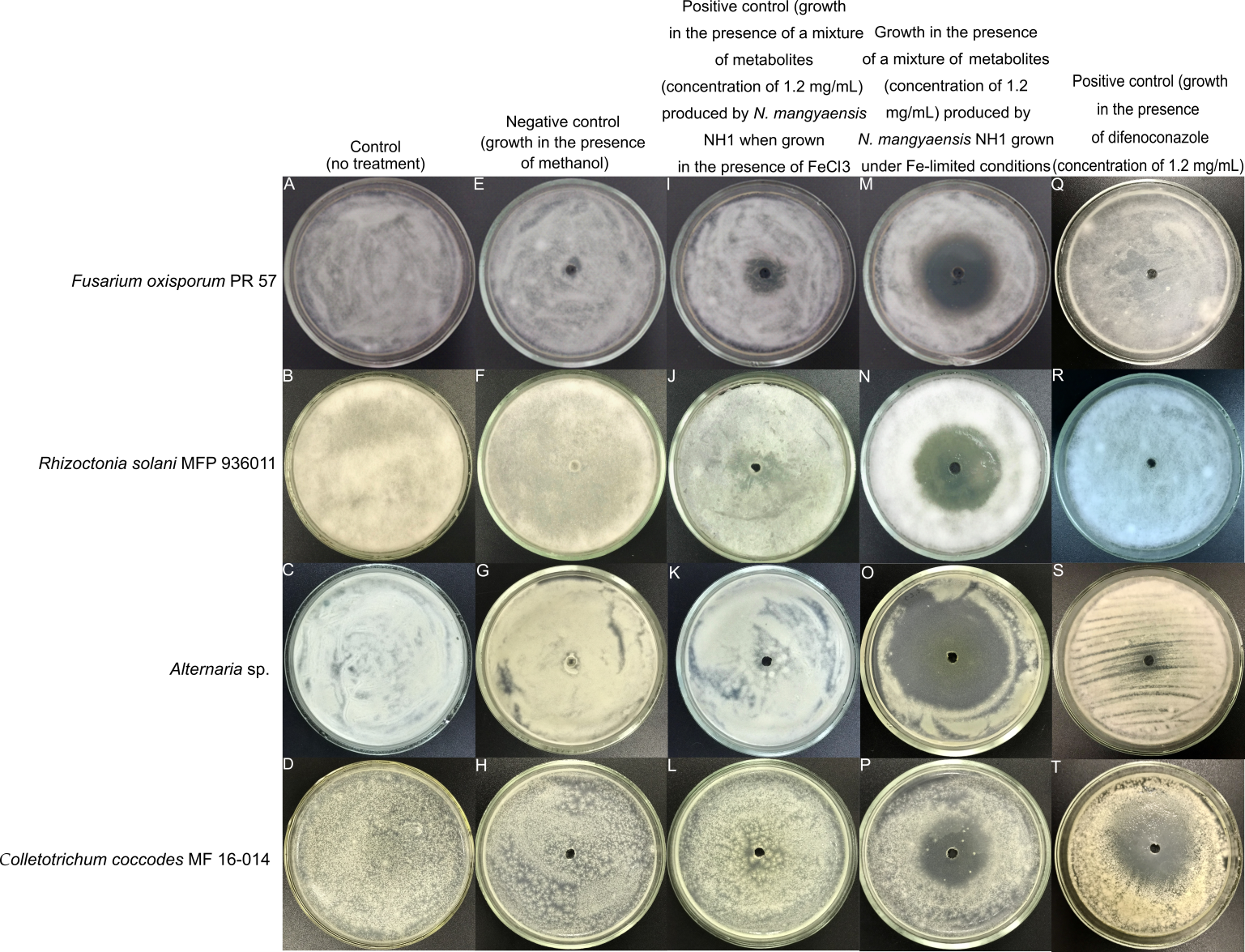
**

**Figure S10**. Antifungal activity of metabolites produced by *N. mangyaensis* strain H1 under Fe-limited conditions against phytopatogenic *Fusarium oxysporum* PR57, *Rhizoctonia solani* MFP 936011, *Colletotrichum coccodes* MF 16-014 and *Alternaria* sp. (A-D), fungal strains without treatment (control); (E-H), negative control - growth of fungal strains in the presence of methanol; (I-L), positive control - growth of fungal strains in the presence of a mixture of metabolites (at a concentration of 1.2 mg/mL) produced by *N. mangyaensis* NH1 grown in the presence of FeCl_3_; (M-P), growth of fungal strains in the presence of a mixture of metabolites from *N. mangyaensis* NH1 (at a concentration of 1.2 mg/mL); (Q-T), positive control (growth in the presence of difenoconazole (concentration of 1.2 mg/mL)). All metabolites were dissolved in the methanol. All experiments were performed in triplicates.


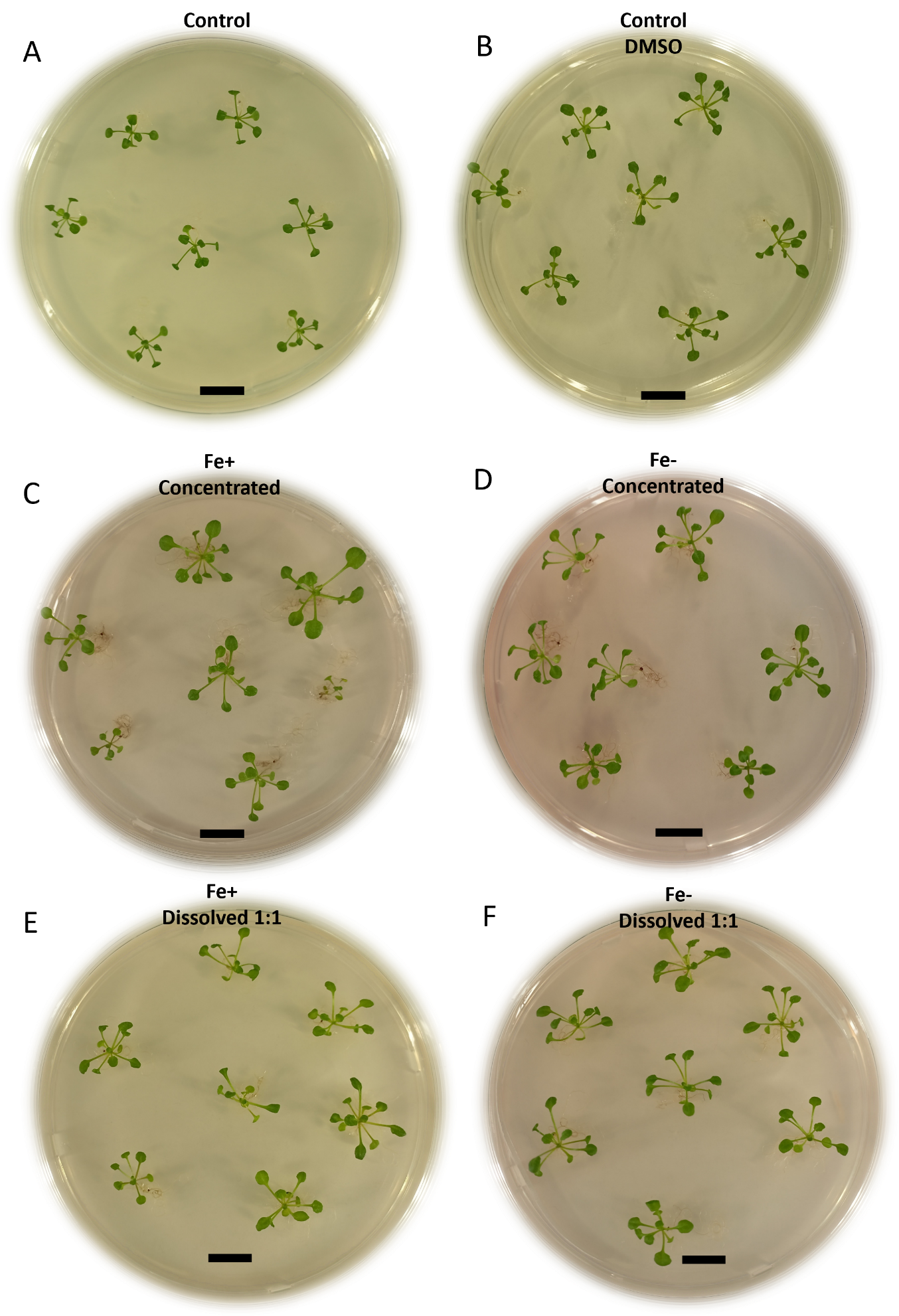


**Figure S11**. Phytotoxic activity of mixture of metabolites produced by *N. mangyaensis* NH1 on *Arabidopsis thaliana* after 7 days. (A), control (no treatment); (B), control (DMSO); treatment with a mixture of metabolites at a concentration of (C), 1.2 mg/mL and (E), 0.6 mg/mL produced by *N. mangyaensis* NH1 grown in the presence of FeCl_3_; treatment with a mixture of metabolites at a concentration of (D), 1.2 mg/mL and (F), 0.6 mg/mL produced by *N. mangyaensis* strain H1 grown under Fe-deficient conditions. Scale bar 10 mm.


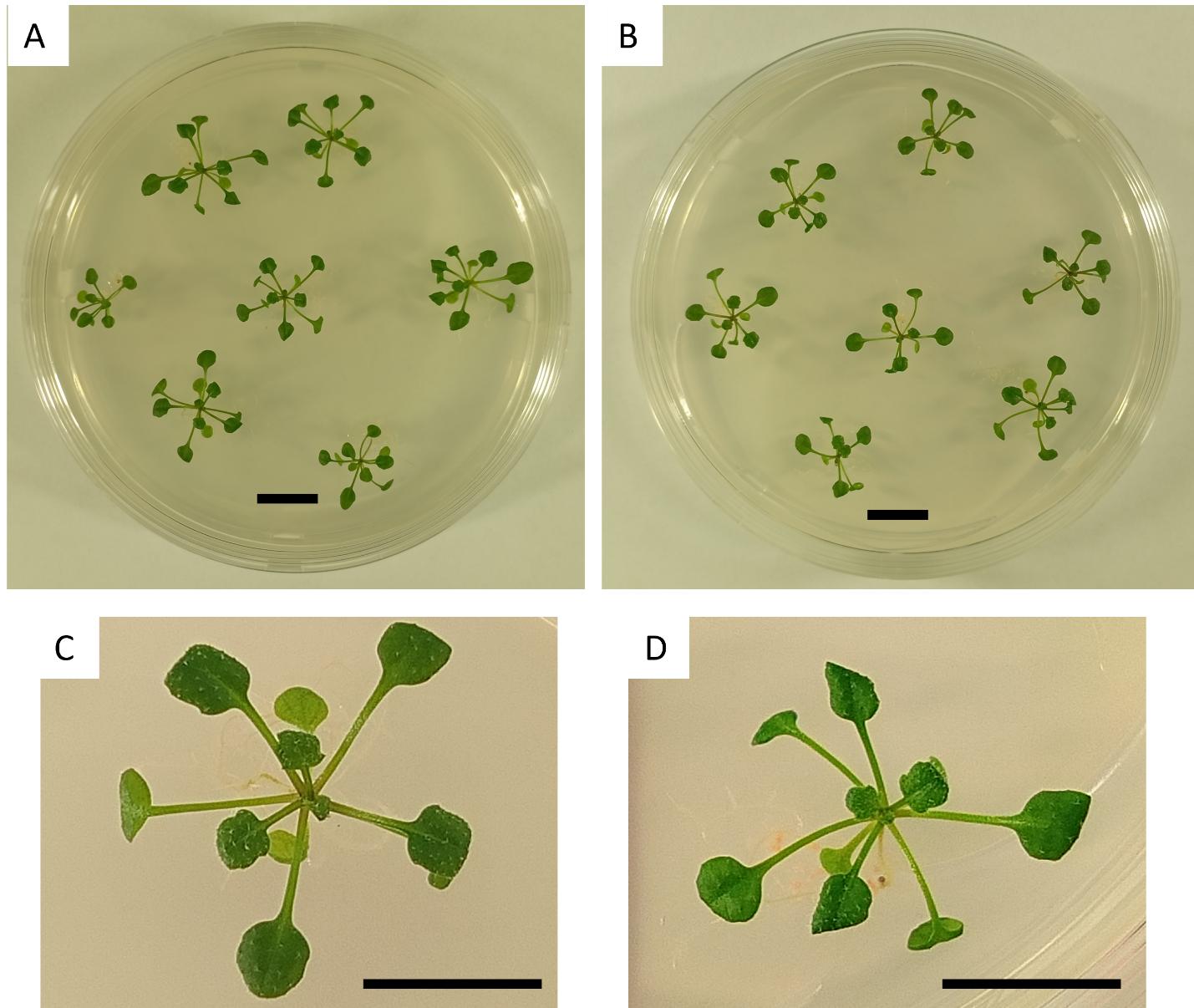


|  |  |
| --- | --- |

**Figure S12.** Plant infection assay of *N. mangyaensis* NH1 on *Arabidopsis thaliana*. (A,C), control, (treated with 1% sucrose); (B,D), *A. thaliana* seedlings treated with *N. mangyaensis* NH1 (1.0, initial optical density). Scale bar 10 mm. All treatments of *A. thaliana* seedlings by NH1 strain were performed in triplicates.


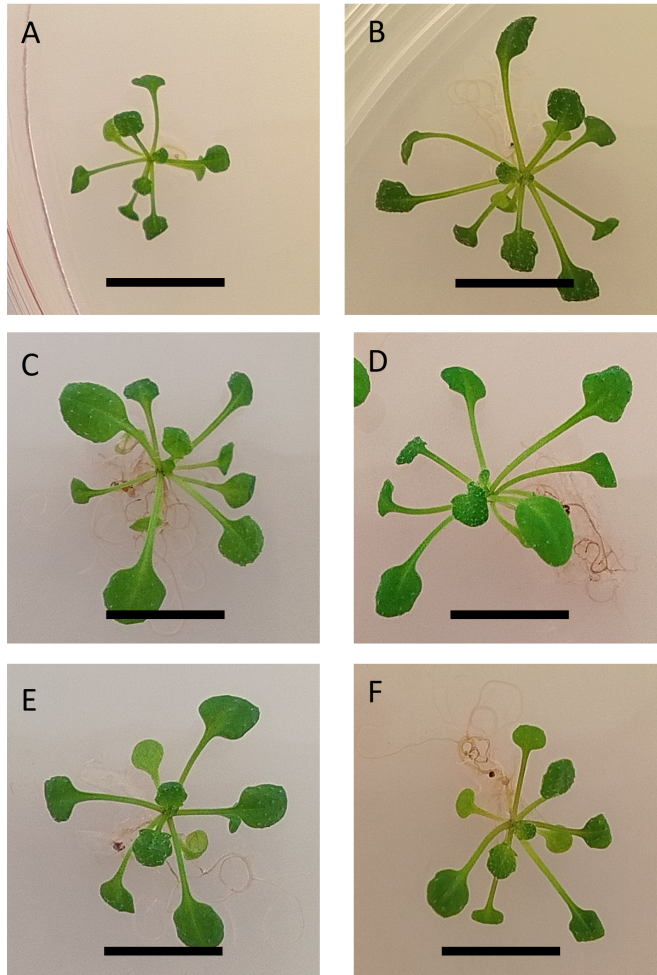


**Figure S13.** Phytotoxicity of a mixture of metabolites produced by *N. mangyaensis* NH1 on *Arabidopsis thaliana* after assessed 7 days experiments. (A), Control (untreated); (B), Control (treated with DMSO); Treatment by a mixture of metabolites in concentration (C), at 1.2 mg/mL and (E), at 0.6 mg/mL derived from the culture of *N. mangyaensis* NH1 grown in the presence of FeCl_3_; Treatment by a mixture of metabolites in concentration (D), at 1.2 mg/mL and (F), at 0.6 mg/mL derived from the culture of *N. mangyaensis* NH1 grown under Fe-deficient conditions. Scale bar 10 mm.
